# Effect of Oral Administration of Metronidazole or Prednisolone on Fecal Microbiota in Dogs

**DOI:** 10.1371/journal.pone.0107909

**Published:** 2014-09-17

**Authors:** Hirotaka Igarashi, Shingo Maeda, Koichi Ohno, Ayako Horigome, Toshitaka Odamaki, Hajime Tsujimoto

**Affiliations:** 1 Department of Veterinary Internal Medicine, Graduate School of Agricultural and Life Science, The University of Tokyo, Tokyo, Japan; 2 Food Science and Technology Institute, Morinaga Milk Industry Co. Ltd., Kanagawa, Japan; University of Illinois, United States of America

## Abstract

Gastrointestinal microbiota have been implicated in the pathogenesis of various gastrointestinal disorders in dogs, including acute diarrhea and chronic enteropathy. Metronidazole and prednisolone are commonly prescribed for the treatment of these diseases; however, their effects on gastrointestinal microbiota have not been investigated. The objective of this study was to evaluate the effects of these drugs on the gastrointestinal microbiota of dogs. Metronidazole was administered twice daily at 12.5 mg/kg to a group of five healthy dogs, and prednisolone at 1.0 mg/kg daily to a second group of five healthy dogs for 14 days. Fecal samples were collected before and after administration (day 0 and 14), and 14 and 28 days after cessation (day 28 and 42). DNA was extracted, and the bacterial diversity and composition of each sample were determined based on 16S ribosomal RNA (rRNA) gene sequences using next-generation sequencing (Illumina MiSeq). In the group administered metronidazole, bacterial diversity indices significantly decreased at day 14, and recovered after the cessation. Principal coordinates analysis and hierarchical dendrogram construction based on unweighted and weighted UniFrac distance matrices revealed that bacterial composition was also significantly altered by metronidazole at day 14 compared with the other time points. The proportions of Bacteroidaceae, Clostridiaceae, Fusobacteriaceae, Lachnospiraceae, Ruminococcaceae, Turicibacteraceae, and Veillonellaceae decreased, while Bifidobacteriaceae, Enterobacteriaceae, Enterococcaceae, and Streptococcaceae increased at day 14 and returned to their initial proportions by day 42. Conversely, no effect of prednisolone was observed on either the bacterial diversity or composition. Reducing pathogenic bacteria such as Fusobacteria and increasing beneficial bacteria such as *Bifidobacterium* through the administration of metronidazole may be beneficial for promoting gastrointestinal health; however, further investigations into the effects on diseased dogs are needed.

## Introduction

Gastrointestinal (GI) microbiota have been shown to play a crucial role in the maintenance of host GI health in humans and dogs [1]–[3]. They form an integral part of the intestinal barrier and protect the host from pathogens through several mechanisms, including colonization resistance and competition for nutrients and mucosal adhesion sites, which physiologically restricts the environment available to invading pathogens [4]. In addition, GI microbiota have enzymes that digest complex carbohydrates from the diet and ferment endogenous products, including sloughed epithelial cells and mucus; this process results in the production of short-chain fatty acids (SCFA), which are used as an energy source for epithelial cell growth and metabolism [5].

Currently, the pathogenic mechanism of inflammatory bowel disease (IBD) in humans is thought to involve an abnormal interaction between commensal microbiota and the GI immune system in genetically predisposed individuals [6]. A similar mechanism is proposed to explain various canine GI disorders [2], [7], and the role of GI microbiota in the pathogenesis of certain canine GI disorders has been reported. Specific pathogens such as enterotoxigenic *Clostridium perfringens*, *Clostridium difficile*, *Campylobacter* spp., and *Salmonella* spp. have been associated with acute diarrhea [8], [9], and are treated with appropriate antibiotics and/or supportive therapy [8]. However, non-specific dysbiosis has been reported in chronic enteropathy (CE) [9]–[13]. Canine CE is commonly treated with dietary management, antibiotics (including metronidazole and tylosin), corticosteroid drugs, or combination of aforementioned treatments [14]–[17]. The disorder is subsequently diagnosed as food-responsive enteropathy (FRE), antibiotics-responsive enteropathy (ARE), or IBD, based on the response to treatment [14].

To date, many studies have characterized the effect of dietary intervention, such as dietary fiber, animal-derived protein, carbohydrates, and synbiotics on GI microbiota in dogs [18]–[21]. Conversely, information regarding the effect of antibiotics on the composition of canine GI microbiota is limited, although it is well known that antibiotics can alter the GI microbiota. One study described the effect of tylosin on jejunal microbiota in healthy dogs, and revealed that the proportions of *Enterococcus*-like organisms, *Pasteurella* spp., and *Dietzia* spp. increased, while Fusobacteria, Bacteroidales, and *Moraxella* decreased during treatment [22]. In contrast, no study has evaluated the effect of metronidazole on GI microbiota in healthy dogs. Furthermore, information regarding steroid therapy is also lacking. Therefore, the objective of the present study was to evaluate the effect of metronidazole or prednisolone on canine GI microbiota using high-throughput 16S ribosomal RNA (rRNA) gene sequencing.

## Materials and Methods

### Ethics statement

The drug administration and fecal sampling were approved by the Animal Care Committee of the University of Tokyo (Approval No. P13-773).

### Animals

In total, 10 healthy beagles were used in the present study, including four females (two intact and two neutered) and six males (four intact and two neutered). Their median age was 49 months (range, 45–127 months), median body weight was 14.15 kg (range, 10.6–17.4 kg), and median body condition score was 5.5 (range, 4–7), based on a 9-point scale [23]. These dogs had no clinical signs of gastrointestinal disease and showed no abnormalities as determined by blood test, fecal examination, and ultrasound. They were not administered any drugs three months prior to the current study. The dogs received a commercial dry food (Hill's prescription diet d/d Rice & Egg, Hill's Pet Nutrition, Inc., Kansas, USA) once a day throughout the study period. According to the manufacturer, this food was composed of 59.5% carbohydrate, 18.2% crude protein, 16.7% crude fat, 4.2% crude ash, and 1.4% crude fiber. The dogs were housed at the same laboratory animal unit in separate pens at the Veterinary Medical Center of the University of Tokyo, and were treated individually.

### Drug administration

Metronidazole was administered orally at 12.5 mg/kg every 12 h to five dogs, and prednisolone at 1.0 mg/kg daily to another five dogs for 14 days. The doses of each drug are equivalent to that used for the treatment of canine CE [16], [24].

### Sample collection

Naturally passed feces were collected from each dog within 10 min of defecation both before and after drug administration (day 0 and 14), and 14 and 28 days after withdrawal (day 28 and 42), and frozen at −80°C until further analysis.

### DNA extraction

Fecal samples (20 mg) were suspended in 450 µl extraction buffer (100 mM Tris/HCl, 40 mM EDTA, pH 9.0), and 50 µl 10% SDS. Glass beads (300 mg, 0.1 mm diameter) and 500 µl buffer-saturated phenol were added to the suspension, and the mixture was vortexed vigorously for 30 s using a FastPrep FP 100A (MP Biomedicals, LLC, Santa Ana, CA, USA) at a power level of 5. After centrifugation at 14,000× *g* for 5 min, 400 µl of the supernatant was extracted with phenol/chloroform, and 250 µl of supernatant was precipitated with propan-2-ol. Purified DNA was rinsed with 300 µl 70% ethanol, and then suspended in 200 µl Tris/EDTA buffer (pH 8.0).

### 16S rRNA gene sequencing

Amplification and sequencing of the V4 region of the bacterial 16S rRNA gene was performed using validated, region-specific bacterial/archaeal primers 515F and 806R, according to previously described methods optimized for the Illumina MiSeq platform (Illumina, Inc., San Diego, CA, USA) [25]. 5′-Barcoded amplicons were generated using TaKaRa Ex Taq HS (Takara Bio Inc., Shiga, Japan). The amplification conditions were as follows: 94°C for 3 min, 25 cycles of PCR (94°C for 45 s, 50°C for 1 min, and 72°C for 1.5 min), and a final elongation step of 72°C for 10 min. The amplicons were pooled in equimolar concentration and sequenced with an Illumina MiSeq platform using MiSeq Reagent Kit v1 (Illumina, Inc.).

Raw 150 bp paired-end sequence reads were combined using the script fastq-join (ea-utils-1.1.2-301.x86_64.rtp: https://code.google.com/p/ea-utils/downloads/list) with the default settings. Further data processing included filtering and denoising by clustering similar sequences with less than 3% dissimilarity using USEARCH v5.2.32 (http://drive5.com/usearch/) [26], and de-novo chimera detection and removal in UCHIME (http://drive5.com/usearch/manual/uchime_algo.html) [27]. 16S rRNA operational taxonomic units (OTUs) were selected from the combined reads using a de-novo OTU picking protocol clustered at 97% identity through the Quantitative Insights Into Microbial Ecology (QIIME) pipeline software version 1.6.0 (http://qiime.org) [25], with USEARCH against the Greengenes database (http://greengenes.secondgenome.com/downloads/database/12_10; Oct. 2012 release). The representative sequences for each OTU were compared with those in the Greengenes database for taxonomy assignment. Of the 494,883 sequences processed, 99.5% (492,438) shared more than 97% sequence identity with a reference sequence. To account for unequal sequencing depth across samples, subsequent analyses were performed on a randomly selected subset of 9,915 or 8,153 sequences per sample for dogs administered metronidazole or prednisolone, respectively.

### Statistical analysis

To estimate bacterial diversity of each sample, three indices—number of OTUs, Shannon index, and Chao1—were calculated and rarefraction curves were depicted using QIIME [28], [29]. Differences in microbial communities among samples were investigated using phylogeny-based unweighted or weighted UniFrac distance matrices, which were calculated using the Greengenes reference tree. Principal coordinates analysis (PCoA) and hierarchical dendrogram construction were performed using QIIME. Differences in microbiota composition between samples obtained at each time point were tested using the one-way analysis of similarity (ANOSIM) function in the statistical software package PRIMER 6 (PRIMER-E Ltd., Luton, UK).

Differences in the bacterial diversity indices and the proportions of bacterial taxa between time points were determined using repeated measures ANOVA or Friedman's test, where appropriate (JMP Pro version 10.0.2, SAS Institute, Cary, NC, USA). Only bacterial taxa that were present in at least three of five dogs (on day 0, 14, 28, or 42) were included in the analysis. A value of *P*<0.05 was considered to be statistically significant for all analyses.

## Results

### Animals

All dogs tolerated the course of metronidazole or prednisolone well, and remained clinically healthy without obvious gastrointestinal side effects (e.g., vomiting and diarrhea) during the study period. Their body weights or body condition scores did not change during the study.

### Characterization of the canine fecal microbiota

**Figure 1:**
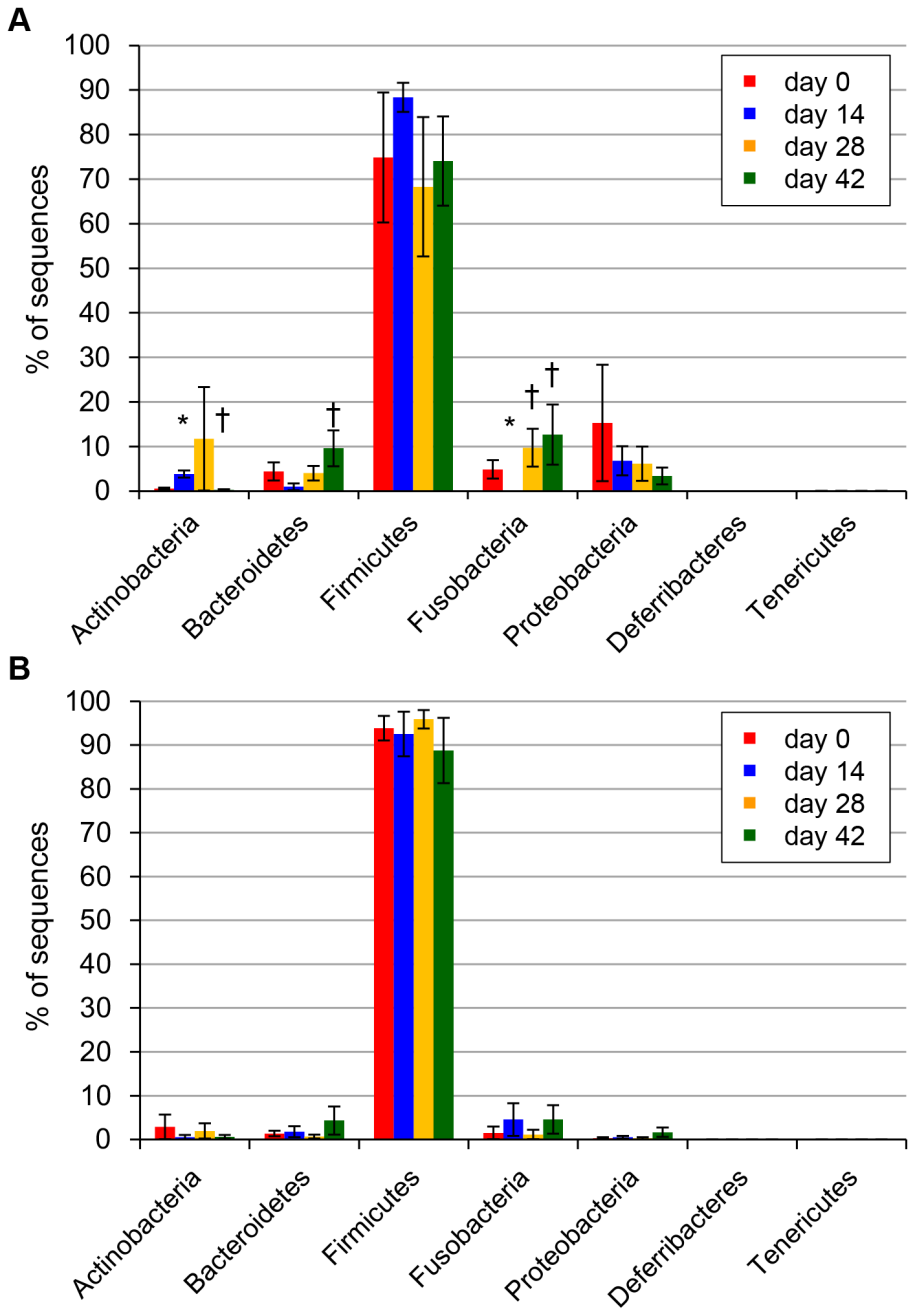
Average proportion of bacterial phyla identified in dogs at each time point. Results of dogs administered metronidazole (A) and prednisolone (B). Error bars represent standard error of the mean. Asterisks indicate statistically significant differences from day 0, and daggers indicate statistically significant differences from day 14 (*P*<0.05).

On day 0, sequences were classified into seven bacterial phyla across all samples (Figure 1). The major bacterial phyla were Firmicutes (84.4% of all sequences), Proteobacteria (7.8%), Fusobacteria (3.2%), Bacteroidetes (2.9%), and Actinobacteria (1.7%). The phyla Deferribacteres and Tenericutes each accounted for <0.1% of all obtained sequencing tags.

### Effect of metronidazole on bacterial diversity indices

**Figure 2:**
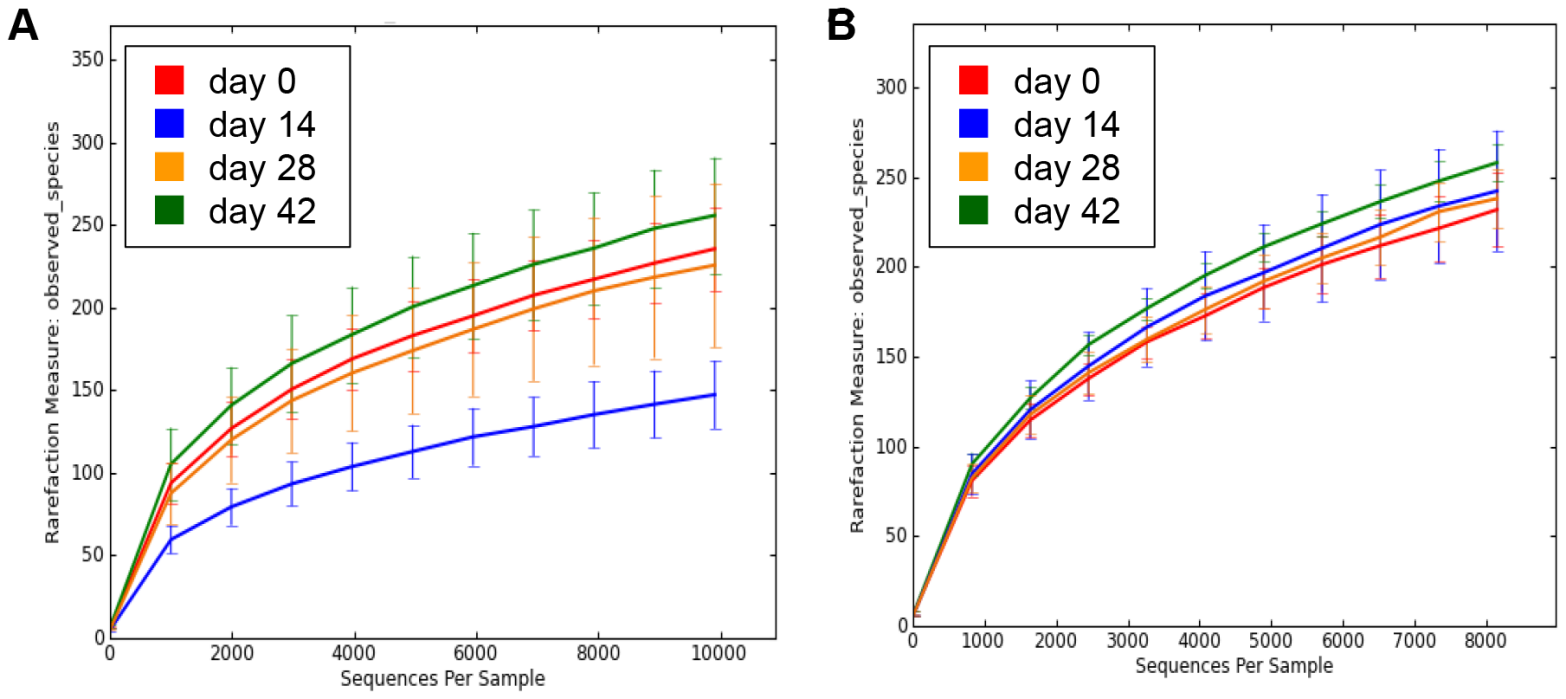
Rarefraction analysis of V4 16S rRNA gene sequences obtained from fecal samples. Results from dogs administered metronidazole (A) and prednisolone (B). Lines represent the average of each time point and the error bars represent standard deviations. This analysis was performed using a randomly selected subset of 9,915 (A) or 8,153 (B) sequences per sample. Operational Taxonomical Units (OTUs) in this analysis were defined by 97–100% similarity.

Figure 2 illustrates the rarefraction curves for each time point. All three bacterial diversity indices significantly decreased at day 14 with metronidazole administration, and subsequently rebounded by day 42 (Table 1).

**Table 1:** Effect of metronidazole and prednisolone on bacterial diversity indices.

	day 0	day 14	day 28	day 42
Metronidazole				
OTU	235.7±28.3	147.4±23.0*	225.9±55.7	256.0±39.1†
Shannon Index	4.37±0.61	3.21±0.65*	3.80±0.83	4.78±0.83†
Chao1	331.8±45.5	234.8±40.3*	323.0±97.2	361.5±49.6†
Prednisolone				
OTU	231.7±23.2	242.1±37.6	237.9±18.7	257.8±11.8
Shannon Index	4.02±0.76	4.23±0.44	4.00±0.19	4.41±0.64
Chao1	364.1±62.3	356.4±42.2	380.0±25.6	387.4±43.7

Data represents mean ± SD.

*Significantly different from day 0 (*P*<0.05).

† Significantly different from day 14 (*P*<0.05).

### Effect of metronidazole on bacterial composition

**Figure 3:**
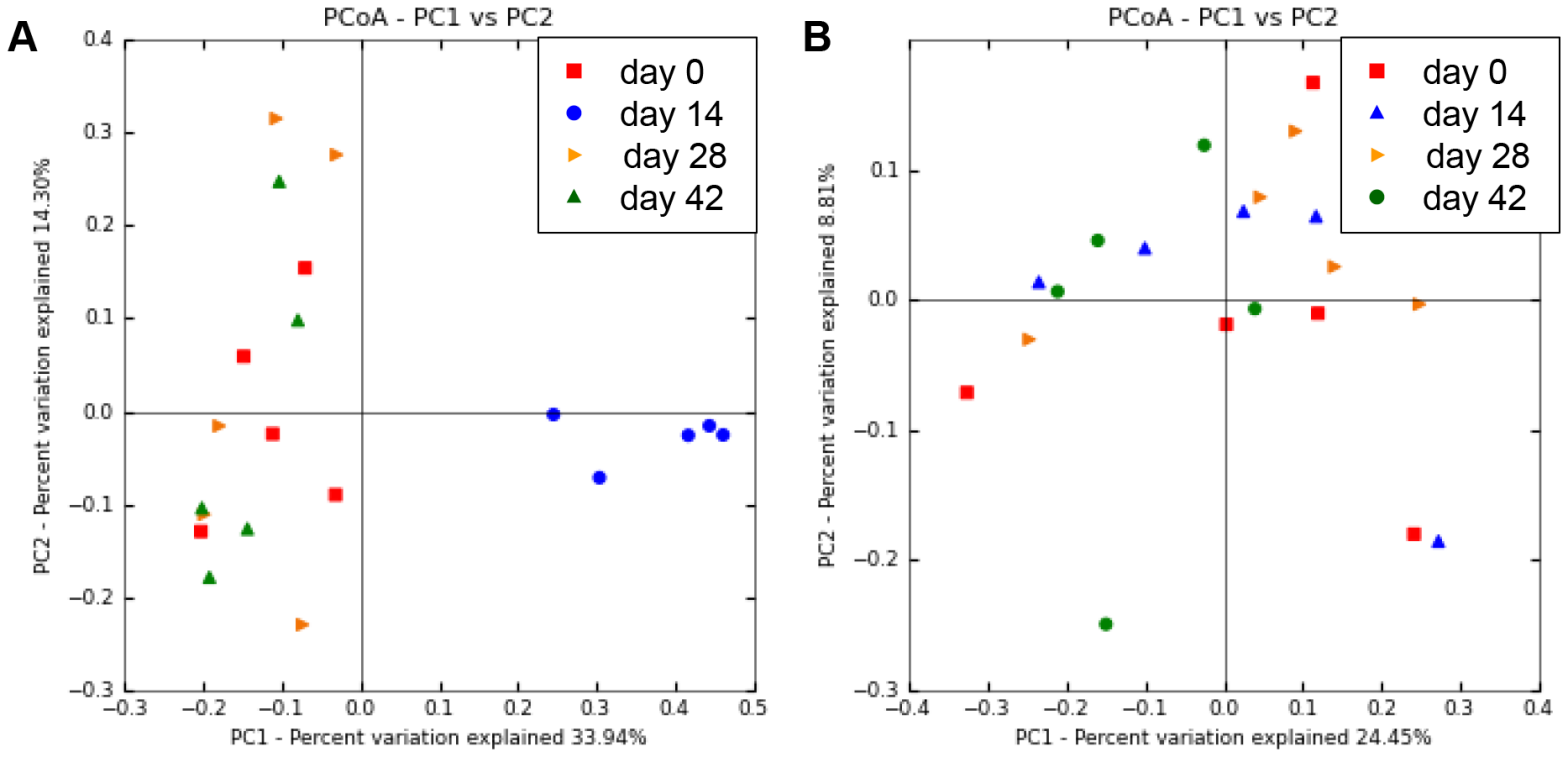
Principal coordinates analysis (PCoA) of V4 16S rRNA genes from canine fecal samples. Figures were calculated using unweighted UniFrac distances. (A) Result of dogs administered metronidazole. Metronidazole-affected samples (blue, day 14) were separated from the other samples, primarily along PCoA axis 1 (accounting for 33.94% of all variability among samples). (B) Result of dogs administered prednisolone. Prednisolone administration did not induce alteration of bacterial composition.

**Figure 4:**
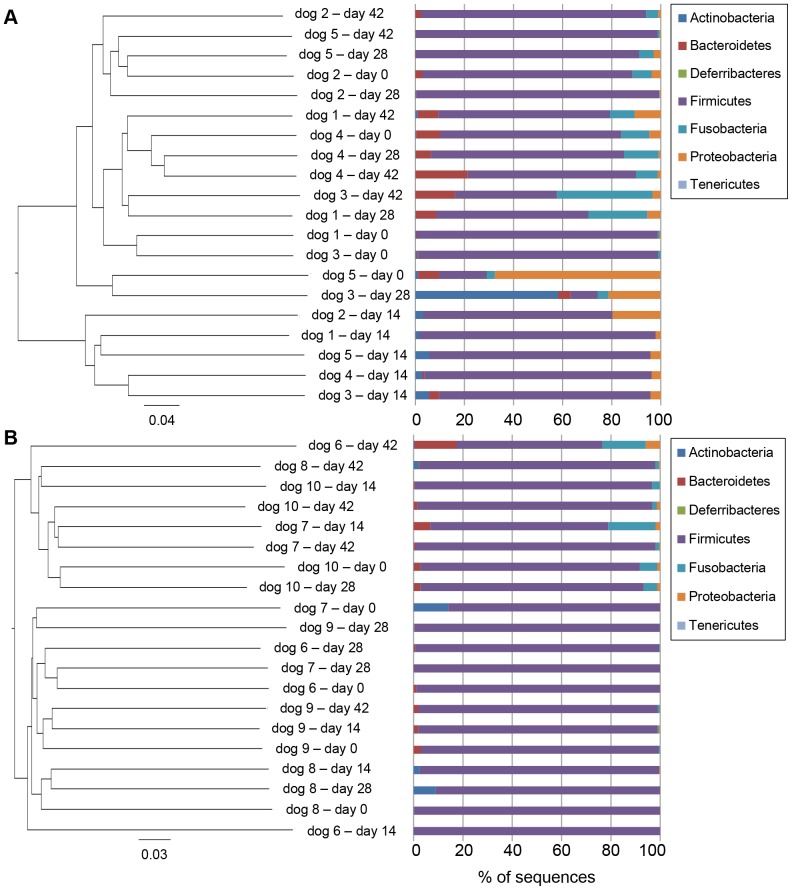
Hierarchical dendrogram and fecal microbial composition of each sample at the phylum level. Figures were constructed using unweighted UniFrac distances. (A) Result of dogs administered metronidazole. This dendrogram showed that the samples obtained at day 14 were clustered. (B) Result of dogs administered prednisolone. No clustering was observed at any time points.

Bacterial composition of fecal microbiota from healthy dogs after metronidazole administration (day 14) was significantly different from the baseline composition (day 0). Furthermore, the microbiota after withdrawal (day 28 and 42) was also different from that of day 14, but relatively similar to that of the baseline. PCoA plots and hierarchical dendrogram based on the unweighted UniFrac distance matrices (Figure 3 and 4) were generated to compare samples at each time point, and showed significant differences between the samples collected at day 14 and all other time points (ANOSIM; global R = 0.428, *P* = 0.001; day 0 vs. day 14, R = 0.926, *P* = 0.008; day 14 vs. day 28, R = 0.944, *P* = 0.008; day 14 vs. day 42, R = 0.956, *P* = 0.008). In contrast, no significant differences were observed between the other time points (ANOSIM; day 0 vs. day 28, R = −0.104, *P* = 0.786; day 0 vs. day 42, R = −0.136, *P* = 0.960; day 28 vs. day 42, R = −0.024, *P* = 0.508). Furthermore, the PCoA plots and hierarchical dendrogram constructed with the weighted UniFrac distance matrices also showed similar results (Figure S1 and S2).

The proportion of the phylum Actinobacteria significantly increased at day 14, while the phylum Fusobacteria was significantly decreased by metronidazole administration (Figure 1). Table 2 summarizes the phylogenic distribution of the most predominant bacterial taxa. In addition, the proportions of all bacterial taxa analyzed in this study was represented in Table S1.

**Table 2:** Relative proportions of the most predominant bacterial taxa in the dogs with metronidazole administration.

	Medians % (min.–max. %) of sequences			
	day 0	day 14	day 28	day 42
Actinobacteria (phylum)	0.38 (0.02–1.20)	3.19* (1.58–5.81)	0.17 (0.03–58.24)	0.03† (0.02–1.01)
Actinobacteria (class)	0.38 (0.02–1.20)	3.19* (1.58–5.81)	0.17 (0.03–58.24)	0.03† (0.02–1.01)
Bifidobacteriales	0.38 (0.01–0.84)	3.18* (1.48–5.71)	0.03† (0.00–0.18)	0.02† (0.00–0.84)
Bifidobacteriaceae	0.37 (0.01–0.84)	3.18* (1.48–5.71)	0.03† (0.00–0.18)	0.02† (0.00–0.84)
*Bifidobacterium*	0.37 (0.01–0.84)	3.18* (1.48–5.71)	0.03† (0.00–0.18)	0.02† (0.00–0.84)
Bacteroidetes	2.84 (0.36–9.82)	0.02 (0.00–4.01)	4.95 (0.27–8.36)	8.27† (0.14–21.26)
Bacteroidia	2.84 (0.36–9.82)	0.02 (0.00–4.01)	4.95 (0.27–8.36)	8.27† (0.14–21.26)
Bacteroidales	2.84 (0.36–9.82)	0.02 (0.00–4.01)	4.95 (0.27–8.36)	8.27† (0.14–21.26)
Bacteroidaceae	1.93 (0.07–6.89)	0.01* (0.00–0.04)	1.39 (0.15–3.17)	5.01† (0.07–7.80)
*Bacteroides*	1.93 (0.07–6.89)	0.01* (0.00–0.04)	1.39 (0.15–3.17)	5.01† (0.07–7.80)
Prevotellaceae	0.87 (0.26–6.91)	0.00 (0.00–4.00)	2.38 (0.04–4.98)	2.54 (0.07–15.80)
*Prevotella*	0.87 (0.26–6.91)	0.00 (0.00–4.00)	2.38 (0.04–4.98)	2.54 (0.07–15.80)
Firmicutes	85.61 (19.42–98.03)	89.92 (77.01–96.23)	78.59 (11.02–99.02)	69.97 (41.54–98.51)
Bacilli	4.66 (0.25–11.90)	68.75* (44.00–80.10)	1.36† (0.73–5.42)	3.66† (1.60–10.72)
Lactobacillales	0.29 (0.05–11.84)	68.74* (43.84–80.07)	0.07† (0.03–5.31)	0.12† (0.03–4.04)
Enterococcaceae	0.01 (0.00–10.59)	2.58 (1.07–8.20)	0.00 (0.00–4.70)	0.00 (0.00–0.01)
Lactobacillaceae	0.28 (0.01–5.04)	10.13 (0.01–66.89)	0.04 (0.01–0.42)	0.05 (0.01–4.00)
*Lactobacillus*	0.13 (0.01–5.02)	6.31 (0.01–66.41)	0.01 (0.00–0.42)	0.02 (0.01–4.00)
Streptococcaceae	0.01 (0.00–0.17)	58.49* (0.69–71.24)	0.03† (0.01–0.13)	0.03† (0.02–0.06)
*Streptococcus*	0.00 (0.00–0.13)	1.16* (0.05–1.61)	0.03 (0.00–0.12)	0.01† (0.01–0.03)
Turicibacterales	2.36 (0.04–5.76)	0.01* (0.00–1.17)	0.74 (0.12–1.73)	3.58† (1.45–6.66)
Turicibacteraceae	2.36 (0.04–5.76)	0.01* (0.00–1.17)	0.74 (0.12–1.73)	3.58† (1.45–6.66)
*Turicibacter*	2.36 (0.04–5.76)	0.01* (0.00–1.17)	0.74 (0.12–1.73)	3.58† (1.45–6.66)
Clostridia	74.50 (5.93–77.43)	3.45* (0.54–7.67)	65.34† (4.62–87.46)	48.75† (32.31–79.63)
Clostridiales	73.61 (5.90–76.87)	2.99* (0.46–7.59)	64.14† (4.59–87.09)	47.97† (32.09–78.95)
Clostridiaceae	40.44 (2.42–51.29)	1.25* (0.02–3.73)	35.99† (1.13–65.48)	20.86† (11.28–49.81)
*Clostridium*	38.73 (2.42–51.27)	0.13* (0.02–2.90)	35.90† (1.11–65.43)	20.52† (10.87–49.78)
Lachnospiraceae	16.11 (1.63–18.98)	0.03* (0.01–0.05)	13.36 (1.37–20.28)	13.64† (9.01–19.60)
*Blautia*	11.01 (0.28–16.44)	0.02* (0.00–0.03)	5.08 (0.70–14.72)	9.21† (6.36–11.46)
*Dorea*	0.47 (0.10–2.57)	0.01* (0.00–0.01)	2.17 (0.02–11.93)	1.38† (0.68–5.11)
Peptococcaceae	2.44 (0.00–3.50)	0.00* (0.00–0.00)	0.12 (0.00–1.61)	0.52 (0.00–2.14)
*Peptococcus*	2.44 (0.00–3.50)	0.00* (0.00–0.00)	0.12 (0.00–1.61)	0.52 (0.00–2.14)
Peptostreptococcaceae	3.37 (0.21–5.85)	0.27 (0.10–4.27)	1.27 (0.32–4.40)	4.09 (1.59–6.61)
Ruminococcaceae	4.51 (0.81–10.16)	0.03* (0.00–0.32)	6.02 (0.97–8.05)	6.49† (5.13–20.97)
*Ruminococcus*	4.26 (0.60–9.97)	0.03* (0.00–0.32)	5.65 (0.76–6.24)	4.32† (2.68–20.86)
Veillonellaceae	0.12 (0.03–0.97)	0.00* (0.00–0.01)	0.40† (0.01–4.90)	1.45† (0.07–2.38)
Erysipelotrichi	10.92 (0.67–20.97)	7.41 (2.93–40.69)	7.83 (0.96–22.78)	7.33 (4.70–26.00)
Erysipelotrichales	10.92 (0.67–20.97)	7.41 (2.93–40.69)	7.83 (0.96–22.78)	7.33 (4.70–26.00)
Erysipelotrichaceae	5.82 (0.46–20.80)	7.05 (2.93–39.39)	5.81 (0.95–17.69)	5.10 (2.98–25.47)
*Allobaculum*	5.74 (0.46–20.74)	6.75 (1.43–39.35)	5.61 (0.91–17.68)	4.64 (2.87–25.39)
Coprobacillaceae	2.65 (0.17–5.32)	0.35 (0.00–1.30)	2.02 (0.01–5.09)	1.71 (0.52–8.07)
*Catenibacterium*	2.37 (0.13–4.79)	0.33 (0.00–1.30)	1.68 (0.00–4.76)	1.64 (0.50–7.60)
Fusobacteria (class)	3.30 (0.88–11.40)	0.00* (0.00–0.01)	5.84† (0.13–23.98)	8.79† (0.87–38.85)
Fusobacteriales	3.30 (0.88–11.40)	0.00* (0.00–0.01)	5.84† (0.13–23.98)	8.79† (0.87–38.85)
Fusobacteriaceae	3.30 (0.88–11.40)	0.00* (0.00–0.01)	5.84† (0.13–23.98)	8.79† (0.87–38.85)
*J2-29*	1.36 (0.01–1.79)	0.00* (0.00–0.00)	0.89 (0.01–2.40)	1.51† (0.03–13.05)
Proteobacteria	3.74 (0.11–67.45)	4.18 (2.15–19.64)	2.90 (0.41–21.19)	1.28 (0.41–10.72)
Gammaproteobacteria	1.38 (0.02–45.79)	3.93 (1.96–18.26)	1.20 (0.18–3.71)	0.70 (0.30–6.94)
Aeromonadales	1.38 (0.00–40.83)	0.35 (0.02–14.51)	0.43 (0.17–3.54)	0.38 (0.07–6.84)
Succinivibrionaceae	1.38 (0.00–40.83)	0.35 (0.02–14.51)	0.43 (0.17–3.54)	0.38 (0.07–6.84)
*Anaerobiospirillum*	1.21 (0.00–40.39)	0.33 (0.02–14.51)	0.22 (0.00–3.52)	0.38 (0.00–6.80)
Enterobacteriales	0.00 (0.00–3.42)	3.58* (1.16–4.10)	0.02 (0.00–0.17)	0.00 (0.00–0.59)
Enterobacteriaceae	0.00 (0.00–3.42)	3.58* (1.16–4.10)	0.02 (0.00–0.17)	0.00 (0.00–0.59)
*Escherichia*	0.00 (0.00–0.47)	2.78* (0.97–3.54)	0.01 (0.00–0.17)	0.00† (0.00–0.53)

Taxa observed in at least three of five dogs with the proportion of >1% (either day 0, 14, 28, or 42) were included in this table.

*Significantly different from day 0 (*P*<0.05).

† Significantly different from day 14 (*P*<0.05).

The increase of Actinobacteria was observed in all five dogs administered metronidazole (median, 0.38% to 3.19%, *P* = 0.025), and the proportion of this phylum subsequently decreased (by day 42) after withdrawal of the drug (3.19% to 0.03%, *P* = 0.041). Two orders within Actinobacteria, Actinomycetales and Bifidobacteriales, were observed, and the genus *Bifidobacterium* accounted for most of the taxa in Actinobacteria, as well as the changes in their proportions (with metronidazole administration, 0.37% to 3.18%, *P* = 0.025; after withdrawal, 3.18% to 0.02%, *P* = 0.022) (Table 2). Conversely, bacterial taxa from other genera in the order Actinomycetales were rare, and did not show any significant change in composition following metronidazole administration (Table 2).

The proportion of bacterial taxa in the phylum Bacteroidetes initially did not show significant alteration after metronidazole administration (2.84% to 0.02%, *P* = 0.180), but it significantly increased by day 42 (0.02% to 8.27%, *P* = 0.041) (Table 2). *Bacteroides* was the predominant genus, and significantly decreased following metronidazole administration (1.93% to 0.01%, *P* = 0.025), followed by an increase by day 42 (0.01% to 5.01%, *P* = 0.017).

Firmicutes was the most abundant phylum observed throughout the study period; its overall microbiota proportion was not significantly changed by metronidazole, but its detailed composition was notably altered (Table 2). Three classes were observed in the Firmicutes: Bacilli, Clostridia, and Erysipelotrichi. The composition of Bacilli significantly increased following metronidazole administration (4.66% to 68.75%, *P* = 0.025), and subsequently decreased after the withdrawal (68.75% to 3.66%, *P* = 0.017); these changes were primarily caused by the changes in the families Lactobacillaceae and Streptococcaceae. In contrast, the class Clostridia showed an inverse trend (with metronidazole administration, 74.50% to 3.45%, *P* = 0.025; after drug withdrawal, 3.45% to 48.75%, *P* = 0.041), driven by changes in the proportional abundance of several bacterial families, including Clostridiaceae, Lachnospiraceae, Peptococcaceae, Ruminococcaceae, and Veillonellaceae. In addition, the class Erysipelotrichi did not show significant alteration.

Within the phylum Fusobacteria, all bacterial taxa observed belonged to the family Fusobacteriaceae (Table 2). The proportion of this family was significantly decreased by metronidazole administration (3.30% to 0.00%, *P* = 0.025), and then increased during the wash-out interval (0.00% to 8.79%, *P* = 0.041). The genus *J2-29* was the major contributor to this proportional change (with metronidazole administration, 1.36% to 0.00%, *P* = 0.025; after drug withdrawal, 0.00% to 1.51%, *P* = 0.017).

The proportion of the phylum Proteobacteria did not show significant alteration (Figure 1). However, the family Enterobacteriaceae, belonging to the class Gammaproteobacteria and order Enterobacteriales, showed a significant increase following metronidazole administration (0.00% to 3.58%, *P* = 0.025), and subsequently showed a tendency to decrease after withdrawal (3.58% to 0.00%, *P* = 0.052) (Table 2). All three genera belonging to Enterobacteriaceae—*Escherichia*, *Morganella*, and *Proteus*—exhibited a similar trend, but other genera did not (Table S1).

### Effect of prednisolone on bacterial diversity indices

Rarefraction curves are depicted in Figure 2. No significant differences in the number of OTUs, Shannon index, and Chao1 metric were observed (Table 1).

### Effect of prednisolone on bacterial composition

Relationships among samples were depicted in PCoA plots and hierarchical dendrogram based on the unweighted UniFrac distance matrices (Figure 3 and 4), and no significant difference in the bacterial composition of fecal microbiota from healthy dogs administered prednisolone was observed throughout the study periods (ANOSIM; global R = −0.039, *P* = 0.770). Furthermore, the PCoA plots and hierarchical dendrogram based on the weighted UniFrac distance matrices showed similar results (Figure S3 and S4), and no statistically significant alteration in bacterial taxa composition was observed (Table S2).

## Discussion

The number and/or composition of canine GI microbiota are affected by a multitude of factors, such as dietary or medical intervention, and are associated with various gastrointestinal disorders [2], [7], [30]. Although metronidazole and/or prednisolone are widely used for the treatment of canine CE, little information regarding their effects on GI microbiota have been reported [15], [17]. Thus, this study was performed to provide basic information on the effects of these drugs on GI microbiota using single breed dogs, and controlling for diet and environment. Our data suggest that metronidazole altered the bacterial composition and reduced bacterial diversity, whereas prednisolone did not; furthermore, the effect of metronidazole was transient, and ceased within 4 weeks of drug cessation. In contrast, a recent study showed that a combination therapy of metronidazole and prednisone administered for 60 days followed by a 30-day washout interval did not alter the proportions of several bacterial groups (as detected with quantitative PCR), including Bacteroidetes, Firmicutes, Fusobacteria, *Bifidobacterium*, Lactobacillus, *Faecalibacterium*, *Escherichia coli*, and *C. perfringens* [31]. The discrepancy between this clinical study and our current research regarding the effect of metronidazole may be due to the disease status of individuals, presence of washout intervals, and/or methodological differences.

Metronidazole is a nitroimidazole antibiotic that prevents bacterial DNA synthesis [32], [33]. Metronidazole has been described as having specific activity against anaerobic bacteria and protozoa; consequently, it has been commonly used for the treatment of intestinal disease in dogs [34]. However, in this study metronidazole did not show specific activity against a particular bacterial group, but affected various bacterial groups; namely, it decreased the proportions of *Bacteroides*, *Turicibacter*, Clostridiales, and Fusobacteriaceae, and increased *Bifidobacterium*, Lactobacillales, and Enterobacteriales. Therefore, these changes in microbiota could have some significance for the treatment of canine CE.

Many bacterial groups in the order Clostridiales significantly decreased following metronidazole administration, but recovered after withdrawal. The primary contributor to the decrease was *Clostridium*. The genus *Clostridium* contains a variety of bacterial species, some of which are considered to be pathogenic, such as *C. perfringens* and *C. difficile* [8], [35], while others such as *Clostridium* cluster XIVa, IV, and XVIII, important producers of SCFA, promote anti-inflammatory effects through the induction of regulatory T cells in the human intestine [36]. Since the proportional changes of each type of *Clostridium* were not investigated in this study, it is difficult to assess the effectiveness of metronidazole on canine CE, and further investigation specifying beyond the genus level is needed. Although pathogenic *Clostridium* are described as commensal in healthy dogs [37], they have been detected at increased levels in the feces of dogs suffering from acute diarrhea [9], [38]; therefore, the decrease of *Clostridium* by metronidazole administration may have a significance in the treatment of acute diarrhea in dogs. Conversely, decreased levels of *Clostridium* were reported in dogs with IBD [11], [12], which indicates that the diminished anti-inflammatory function provided by *Clostridium* might play a role in the pathogenesis of canine IBD. Metronidazole also reduced other bacterial groups of Clostridiales, including *Faecalibacterium*, *Ruminococcus*, and Lachnospiraceae, which are also members of *Clostridium* clusters IV and XIVa [7], [39], [40].

In addition to these groups, Fusobacteria were also decreased by metronidazole administration. Fusobacteriaceae comprises highly heterogeneous species, some of which exhibit a number of pathogenic traits, and are associated with human IBD [41], [42]; however, a decrease in Fusobacteria was reported in dogs with IBD or CE, while increased proportions were observed in dogs with acute hemorrhagic diarrhea [9]–[12]. Furthermore, a randomized-controlled trial of canine IBD treatment revealed no significant differences in clinical outcomes between dogs treated with prednisone alone versus prednisone combined with metronidazole [16]. Taken together, although dysbiosis in dogs with FRE or ARE has not been well characterized, the use of metronidazole in the treatment of canine CE, particularly IBD, seems to be ineffective in these respects.

Metronidazole administration also resulted in an observed increase of beneficial bacteria, such as *Bifidobacterium*, which is consistent with a previous study using healthy rats as the experimental model [43]. *Bifidobacterium* has also been reported to lower intestinal pH through increasing fermentation products, and to modulate the intestinal immune system [44], [45]; thus it is commonly prescribed as a probiotic in both human and veterinary medicine [46], [47]. In addition, metronidazole was also shown to increase the proportions of Lactobacillales, including *Enterococcus*, *Lactobacillus*, and *Streptococcus*, which are also important producers of SCFA, and are commonly used as probiotics both in human and veterinary medicine [20], [48], [49]. Therefore, the increase of these bacterial groups caused by metronidazole might be significant in the treatment of canine CE. To date, information is limited regarding the specific proportions of these bacterial groups needed to produce probiotic effects in dogs; furthermore, the type of samples, methodological difference in DNA extraction, analyzed regions of 16S rRNA genes, and sequencing platform interfere with the interpretation and comparison of bacterial composition data using 16S rRNA gene sequencing analysis [50], [51]. Therefore, it is difficult to determine whether this increase has clinical significance, and further investigations comparing the effects of metronidazole and other various probiotics, together with an evaluation of the effects on GI microbiota in canine CE patients, are needed.

Enterobacteriales, within the Gammaproteobacteria, were also increased by metronidazole administration, and the majority of these sequences were *Escherichia*. Adherent invasive *E. coli* (AIEC) has been associated with the pathogenesis of Crohn's disease [52], and is also responsible for granulomatous colitis in boxer dogs (GCB: also referred to as histiocytic ulcerative colitis), which is a particular form of canine ARE [53]. Furthermore, proportions of *Escherichia*, Enterobacteriaceae, or Proteobacteria were observed to increase in dogs suffering from canine IBD [12], [13]. The species composition of *Escherichia* was not investigated in this study; therefore, further investigations are needed to determine the species affected by metronidazole, and their significance in the treatment of canine CE.

Interestingly, the effects of metronidazole were transient, and ceased following drug withdrawal in this study. These results are partly in line with previous studies: Dion et al. reported the bacterial recolonization of the colon within 6 days of drug cessation, and Abujamel et al. showed that the fecal concentration of the drug became undetectable within a few days after withdrawal [54], [55]. In addition, recurrence of *C. difficile* or *Helicobacter pylori* infection after cessation of medication (including metronidazole) has been a continual problem in human treatment [56], [57]. Thus, the antimicrobial effect of metronidazole ceases quickly after treatment cessation. Studies in humans have showed that, in general, fecal microbiota are resilient to short-term antibiotic therapy; subsequently, the bacterial composition stabilize several months after resilience, but it is different from that before antibiotics administration [58]–[60]. Similar results were also observed in a study of the effects of tylosin on the GI microbiota of healthy dogs; following 14 days of tylosin, the bacterial composition did not recover to their starting state, and their responses to tylosin were highly individualized [22]. Since our observation only extended to 4 weeks after drug withdrawal, that the long-term resilience to treatment was unclear. However, the proportions of some bacterial groups at day 42 were not completely equivalent to that observed at day 0, such as Clostridiales and Fusobacteria (median % sequences at day 0 and 42, 73.61 and 47.97, and 3.30 and 8.79, respectively); in addition, inter-individual response differences were also observed. For example, Clostridia taxa were present in dog 3 at 5.90% at day 0, decreased to 5.50% by day 14, and rebounded to 74.86% by day 42; however, at day 0, Clostridia was present in dog 5 at 74.76%, decreased to 0.48% by day 14, and increased to 32.01% by day 42. In humans, antibiotics are known to disrupt the microbial ecosystem, and the responses to disruptions are individualized and influenced by prior exposure to the same antibiotics [61]. The results of this study indicate that the use of metronidazole in dogs also disrupts the microbiota, and the response after withdrawal is individualized; therefore, further investigations into the long-term effects of these treatments on dogs are warranted.

Notably, metronidazole reduced bacterial diversity indices in the present study. Decreases in microbiota of bacterial diversity have great importance in humans. The use of antibiotics often results in antibiotic-associated diarrhea (AAD), which is due to the disruption of the GI microbial ecosystem and subsequent overgrowth of pathogenic species such as *C. difficile* [62]. Information regarding AAD in dogs is limited; however, it often occurs empirically, and occasionally results in the development of fatal colitis [63]. Interestingly, no dogs receiving metronidazole in this study showed any clinical signs of AAD during the study period, despite the reduction in bacterial diversity. Since the pathogenesis of AAD in dogs has not been investigated, the clinical relevance of the reduction in bacterial diversity indices observed in this study is unclear. As described earlier, antibiotics are useful in the treatment of various GI disorders, including acute diarrhea associated with specific pathogens, and ARE [7], [8]. Moreover, metronidazole has been used as a preferred treatment for AAD and *C. difficile* infections in humans [64]. Therefore, investigations into the pathogenesis of AAD and ARE may provide insight into the significance of the microbiota diversity reduction by metronidazole observed here.

One concerning result was the increase of some bacterial groups by metronidazole, including Enterobacteriaceae, *Enterococcus*, and *Streptococcus*. This may be due to the occurrence of nosocomial or opportunistic infection with antimicrobial resistance [65], [66]. Metronidazole is often prescribed in combination with immunomodulatory drugs such as prednisolone or cyclosporine A for the treatment of canine CE [15], [67]; therefore, caution is warranted when using these together. Since the antimicrobial resistance of these bacterial groups was not examined in the current study, it is unclear whether the resistance against metronidazole was natively possessed or induced by drug administration. Therefore, further investigation into this observation is needed.

Since GI microbiota constitutively interact with the mucosal immune system [36], [68], [69], we also evaluated whether the microbiota was altered by this immunomodulation. In contrast to metronidazole, prednisolone did not induce any change in microbiota in this study. The mechanism underlying the anti-inflammatory effects of corticosteroid comprises various pathways, but it does not account for the role of microbiota [70]; thus, the results of the present study are not surprising. However, since we administered only 1 mg/kg daily of prednisolone, the effect of high-dose prednisolone (i.e., 2–4 mg/kg daily), which is occasionally used for the treatment of canine IBD [14], [15], [67], was undetermined. High-dose corticosteroid can damage the intestinal mucosal barrier [71], which could lead to harmful interactions between mucosal immunity and luminal microbiota.

One most critical limitation was the small number of dogs enrolled in this study. As the large inter-individual and intra-individual temporal variations in fecal microbiota has been reported [72], individualized variations in the changes in the proportions of several bacterial taxa were observed in this study. However, a clear tendency was observed that the bacterial diversity indices, composition of microbiota, and proportions of several bacterial taxa significantly altered after metronidazole administration (day 14) and subsequently returned by withdrawal in all 5 dogs. Furthermore, samples obtained at day 0, 28, and 42 from dogs administered metronidazole did not show significant difference in the bacterial diversity indices, PCoA plots, dendrogram analysis, or proportions of specific bacterial taxa. Moreover, samples of dogs administered prednisolone did not show any significant alteration during the study period. Therefore, we consider that these findings are apparently induced by metronidazole administration. Further investigations using dogs with CE in a larger sample size are warranted.

In summary, we characterized the effects of metronidazole or prednisolone on canine fecal microbiota. Changes in bacterial proportions in some bacterial groups caused by metronidazole were identified, but it is unclear whether these are correlated with clinical outcomes. Therefore, future investigations should address these research questions using dogs with CE, including FRE, ARE, and IBD.

## Supporting Information

Figure S1 **Principal coordinates analysis (PCoA) of weighted UniFrac distances of 16S rRNA genes in dogs administered metronidazole.** Metronidazole-affected samples (blue, day 14) were separated from other samples, primarily along PCoA axis 1 (accounting for 43.73% of all variability among samples).
(TIF)

Figure S2 **Hierarchical dendrogram based on weighted UniFrac distances of 16S rRNA genes and fecal microbial composition of each sample at phylum level in dogs administered metronidazole.** This dendrogram showed that the samples obtained at day 14 were clustered.
(TIF)

Figure S3 **PCoA plots of weighted UniFrac distances of 16S rRNA genes in dogs administered prednisolone.** No clustering was observed at any time points.
(TIF)

Figure S4 **Hierarchical dendrogram based on weighted UniFrac distances of 16S rRNA genes and fecal microbial composition of each sample at phylum level in dogs administered prednisolone.** This dendrogram showed that groups of samples at each time point were not clustered.
(TIF)

Table S1 **Relative proportions of bacterial taxa in dogs administered metronidazole.**
(PDF)

Table S2 **Relative proportions of bacterial taxa in dogs administered prednisolone.**
(PDF)

## References

[1] Hooper LV, Wong MH, Thelin A, Hansson L, Falk PG, et al (2001) Molecular analysis of commensal host-microbial relationships in the intestine. Science 291: 881–884. 11157169 10.1126/science.291.5505.881

[2] Hooda S, Minamoto Y, Suchodolski JS, Swanson KS (2012) Current state of knowledge: the canine gastrointestinal microbiome. Anim Health Res Rev 13: 78–88. 22647637 10.1017/S1466252312000059

[3] Mackie RI, Sghir A, Gaskins HR (1999) Developmental microbial ecology of the neonatal gastrointestinal tract. Am J Clin Nutr 69: 1035S–1045S. 10232646 10.1093/ajcn/69.5.1035s

[4] Kanauchi O, Matsumoto Y, Matsumura M, Fukuoka M, Bamba T (2005) The beneficial effects of microflora, especially obligate anaerobes, and their products on the colonic environment in inflammatory bowel disease. Curr Pharm Des 11: 1047–1053. 15777254 10.2174/1381612053381675

[5] Sunvold GD, Fahey GC Jr, Merchen NR, Reinhart GA (1995) In vitro fermentation of selected fibrous substrates by dog and cat fecal inoculum: influence of diet composition on substrate organic matter disappearance and short-chain fatty acid production. J Anim Sci 73: 1110–1122. 7628955 10.2527/1995.7341110x

[6] Packey CD, Sartor RB (2009) Commensal bacteria, traditional and opportunistic pathogens, dysbiosis and bacterial killing in inflammatory bowel diseases. Curr Opin Infect Dis 22: 292–301. 19352175 10.1097/QCO.0b013e32832a8a5d PMC2763597

[7] Suchodolski JS (2011) Companion animals symposium: microbes and gastrointestinal health of dogs and cats. J Anim Sci 89: 1520–1530. 21075970 10.2527/jas.2010-3377 PMC7199667

[8] Marks SL, Rankin SC, Byrne BA, Weese JS (2011) Enteropathogenic bacteria in dogs and cats: diagnosis, epidemiology, treatment, and control. J Vet Intern Med 25: 1195–1208. 22092607 10.1111/j.1939-1676.2011.00821.x

[9] Suchodolski JS, Markel ME, Garcia-Mazcorro JF, Unterer S, Heilmann RM, et al (2012) The fecal microbiome in dogs with acute diarrhea and idiopathic inflammatory bowel disease. PLOS ONE 7: e51907. 23300577 10.1371/journal.pone.0051907 PMC3530590

[10] Allenspach K, House A, Smith K, McNeill FM, Hendricks A, et al (2010) Evaluation of mucosal bacteria and histopathology, clinical disease activity and expression of Toll-like receptors in German shepherd dogs with chronic enteropathies. Vet Microbiol 146: 326–335. 20615633 10.1016/j.vetmic.2010.05.025

[11] Suchodolski JS, Dowd SE, Wilke V, Steiner JM, Jergens AE (2012) 16S rRNA gene pyrosequencing reveals bacterial dysbiosis in the duodenum of dogs with idiopathic inflammatory bowel disease. PLOS ONE 7: e39333. 22720094 10.1371/journal.pone.0039333 PMC3376104

[12] Suchodolski JS, Xenoulis PG, Paddock CG, Steiner JM, Jergens AE (2010) Molecular analysis of the bacterial microbiota in duodenal biopsies from dogs with idiopathic inflammatory bowel disease. Vet Microbiol 142: 394–400. 19959301 10.1016/j.vetmic.2009.11.002

[13] Xenoulis PG, Palculict B, Allenspach K, Steiner JM, Van House AM, et al (2008) Molecular-phylogenetic characterization of microbial communities imbalances in the small intestine of dogs with inflammatory bowel disease. FEMS Microbiol Ecol 66: 579–589. 18647355 10.1111/j.1574-6941.2008.00556.x

[14] Allenspach K, Wieland B, Gröne A, Gaschen F (2007) Chronic enteropathies in dogs: evaluation of risk factors for negative outcome. J Vet Intern Med 21: 700–708. 17708389 10.1892/0891-6640(2007)21[700:ceideo]2.0.co;2

[15] Craven M, Simpson JW, Ridyard AE, Chandler ML (2004) Canine inflammatory bowel disease: retrospective analysis of diagnosis and outcome in 80 cases (1995–2002). J Small Anim Pract 45: 336–342. 15266855 10.1111/j.1748-5827.2004.tb00245.x

[16] Jergens AE, Crandell J, Morrison JA, Deitz K, Pressel M, et al (2010) Comparison of oral prednisone and prednisone combined with metronidazole for induction therapy of canine inflammatory bowel disease: a randomized-controlled trial. J Vet Intern Med 24: 269–277. 20051005 10.1111/j.1939-1676.2009.0447.x

[17] García-Sancho M, Rodríguez-Franco F, Sainz A, Mancho C, Rodríguez A (2007) Evaluation of clinical, macroscopic, and histopathologic response to treatment in nonhypoproteinemic dogs with lymphocytic-plasmacytic enteritis. J Vet Intern Med 21: 11–17. 17338144 10.1892/0891-6640(2007)21[11:eocmah]2.0.co;2

[18] Middelbos IS, Vester Boler BM, Qu A, White BA, Swanson KS, et al (2010) Phylogenetic characterization of fecal microbial communities of dogs fed diets with or without supplemental dietary fiber using 454 pyrosequencing. PLOS ONE 5: e9768. 20339542 10.1371/journal.pone.0009768 PMC2842427

[19] Hang I, Rinttila T, Zentek J, Kettunen A, Alaja S, et al (2012) Effect of high contents of dietary animal-derived protein or carbohydrates on canine faecal microbiota. BMC Vet Res 8: 90. 22735212 10.1186/1746-6148-8-90 PMC3464166

[20] Garcia-Mazcorro JF, Lanerie DJ, Dowd SE, Paddock CG, Grützner N, et al (2011) Effect of a multi-species synbiotic formulation on fecal bacterial microbiota of healthy cats and dogs as evaluated by pyrosequencing. FEMS Microbiol Ecol 78: 542–554. 22067056 10.1111/j.1574-6941.2011.01185.x

[21] Beloshapka AN, Dowd SE, Suchodolski JS, Steiner JM, Duclos L, et al (2013) Fecal microbial communities of healthy adult dogs fed raw meat-based diets with or without inulin or yeast cell wall extracts as assessed by 454 pyrosequencing. FEMS Microbiol Ecol 84: 532–541. 23360519 10.1111/1574-6941.12081

[22] Suchodolski JS, Dowd SE, Westermarck E, Steiner JM, Wolcott RD, et al (2009) The effect of the macrolide antibiotic tylosin on microbial diversity in the canine small intestine as demonstrated by massive parallel 16S rRNA gene sequencing. BMC Microbiol 9: 210. 19799792 10.1186/1471-2180-9-210 PMC2759960

[23] Baldwin K, Bartges J, Buffington T, Freeman LM, Grabow M, et al (2010) AAHA nutritional assessment guidelines for dogs and cats. J Am Anim Hosp Assoc 46: 285–296. 20610704 10.5326/0460285

[24] Münster M, Hörauf A, Bilzer T (2006) Assessment of disease severity and outcome of dietary, antibiotic, and immunosuppressive interventions by use of the canine IBD activity index in 21 dogs with chronic inflammatory bowel disease. Berl Munch Tierarztl Wochenschr 119: 493–505. 17172138

[25] Caporaso JG, Lauber CL, Walters WA, Berg-Lyons D, Huntley J, et al (2012) Ultra-high-throughput microbial community analysis on the Illumina HiSeq and MiSeq platforms. ISME J 6: 1621–1624. 22402401 10.1038/ismej.2012.8 PMC3400413

[26] Edgar RC (2010) Search and clustering orders of magnitude faster than BLAST. Bioinformatics 26: 2460–2461. 20709691 10.1093/bioinformatics/btq461

[27] Edgar RC, Haas BJ, Clemente JC, Quince C, Knight R (2011) UCHIME improves sensitivity and speed of chimera detection. Bioinformatics 27: 2194–2200. 21700674 10.1093/bioinformatics/btr381 PMC3150044

[28] Chao A (1987) Estimating the population size for capture-recapture data with unequal catchability. Biometrics 43: 783–791. 3427163

[29] Shannon CE (1948) A mathematical theory of communication. Bell Syst Tech J 379–656, 379-423, 623-656.

[30] Kerr KR, Beloshapka AN, Swanson KS (2013) 2011 and 2012 Early Careers Achievement Awards: use of genomic biology to study companion animal intestinal microbiota. J Anim Sci 91: 2504–2511. 23482581 10.2527/jas.2012-6225

[31] Rossi G, Pengo G, Caldin M, Palumbo Piccionello A, Steiner JM, et al (2014) Comparison of Microbiological, Histological, and Immunomodulatory Parameters in Response to Treatment with Either Combination Therapy with Prednisone and Metronidazole or Probiotic VSL#3 Strains in Dogs with Idiopathic Inflammatory Bowel Disease. PLOS ONE 9: e94699. 24722235 10.1371/journal.pone.0094699 PMC3983225

[32] Roe FJ (1977) Metronidazole: review of uses and toxicity. J Antimicrob Chemother 3: 205–212. 559669 10.1093/jac/3.3.205

[33] Upcroft P, Upcroft JA (2001) Drug targets and mechanisms of resistance in the anaerobic protozoa. Clin Microbiol Rev 14: 150–164. 11148007 10.1128/CMR.14.1.150-164.2001 PMC88967

[34] Jergens AE (1994) Rational use of antimicrobials for gastrointestinal disease in small animals. J Am Anim Hosp Assoc 30: 123–131.

[35] Borriello SP (1995) Clostridial disease of the gut. Clin Infect Dis 20: S242–S250. 7548565 10.1093/clinids/20.supplement_2.s242

[36] Atarashi K, Tanoue T, Oshima K, Suda W, Nagano Y, et al (2013) Treg induction by a rationally selected mixture of Clostridia strains from the human microbiota. Nature 500: 232–236. 23842501 10.1038/nature12331

[37] Marks SL, Kather EJ (2003) Bacterial-associated diarrhea in the dog: a critical appraisal. Vet Clin North Am Small Anim Pract 33: 1029–1060. 14552160 10.1016/s0195-5616(03)00091-3

[38] Cave NJ, Marks SL, Kass PH, Melli AC, Brophy MA (2002) Evaluation of a routine diagnostic fecal panel for dogs with diarrhea. J Am Vet Med Assoc 221: 52–59. 12420824 10.2460/javma.2002.221.52

[39] Sokol H, Pigneur B, Watterlot L, Lakhdari O, Bermúdez-Humarán LG, et al (2008) Faecalibacterium prausnitzii is an anti-inflammatory commensal bacterium identified by gut microbiota analysis of Crohn disease patients. Proc Natl Acad Sci USA 105: 16731–16736. 18936492 10.1073/pnas.0804812105 PMC2575488

[40] Liu C, Finegold SM, Song Y, Lawson PA (2008) Reclassification of *Clostridium coccoides*, *Ruminococcus hansenii*, *Ruminococcus hydrogenotrophicus*, *Ruminococcus luti*, *Ruminococcus productus* and *Ruminococcus schinkii* as *Blautia coccoides* gen. nov., comb. nov., *Blautia hansenii* comb. nov., *Blautia hydrogenotrophica* comb. nov., *Blautia luti* comb. nov., *Blautia producta* comb. nov., *Blautia schinkii* comb. nov. and description of *Blautia wexlerae* sp. nov., isolated from human faeces. Int J Syst Evol Microbiol 58: 1896–1902. 18676476 10.1099/ijs.0.65208-0

[41] Allen-Vercoe E, Strauss J, Chadee K (2011) *Fusobacterium nucleatum*: an emerging gut pathogen? Gut Microbes 2: 294–298. 22067936 10.4161/gmic.2.5.18603

[42] Citron DM (2002) Update on the taxonomy and clinical aspects of the genus fusobacterium. Clin Infect Dis 35: S22–S27. 12173104 10.1086/341916

[43] Vasquez N, Suau A, Magne F, Pochart P, Pélissier MA (2009) Differential effects of *Bifidobacterium pseudolongum* strain Patronus and metronidazole in the rat gut. Appl Environ Microbiol 75: 381–386. 19028910 10.1128/AEM.01731-08 PMC2620693

[44] Tanabe S, Kinuta Y, Saito Y (2008) *Bifidobacterium infantis* suppresses proinflammatory interleukin-17 production in murine splenocytes and dextran sodium sulfate-induced intestinal inflammation. Int J Mol Med 22: 181–185. 18636171

[45] Jiang T, Savaiano DA (1997) Modification of colonic fermentation by bifidobacteria and pH in vitro. Impact on lactose metabolism, short-chain fatty acid, and lactate production. Dig Dis Sci 42: 2370–2377. 9398819 10.1023/a:1018895524114

[46] Vieira AT, Teixeira MM, Martins FS (2013) The Role of Probiotics and Prebiotics in Inducing Gut Immunity. Front Immunol 4: 445. 24376446 10.3389/fimmu.2013.00445 PMC3859913

[47] Chrzastowska M, Kander M, Depta A (2009) Prospects for the use of probiotic bacteria in the treatment of gastrointestinal diseases in dogs. Pol J Vet Sci 12: 279–284. 19645362

[48] Gupta V, Garg R (2009) Probiotics. Indian J Med Microbiol 27: 202–209. 19584499 10.4103/0255-0857.53201

[49] Hempel S, Newberry SJ, Maher AR, Wang Z, Miles JN, et al (2012) Probiotics for the prevention and treatment of antibiotic-associated diarrhea: a systematic review and meta-analysis. JAMA 307: 1959–1969. 22570464 10.1001/jama.2012.3507

[50] Kennedy NA, Walker AW, Berry SH, Duncan SH, Farquarson FM, et al (2014) The Impact of Different DNA Extraction Kits and Laboratories upon the Assessment of Human Gut Microbiota Composition by 16S rRNA Gene Sequencing. PLOS ONE 9: e88982. 24586470 10.1371/journal.pone.0088982 PMC3933346

[51] Claesson MJ, Wang Q, O'Sullivan O, Greene-Diniz R, Cole JR, et al (2010) Comparison of two next-generation sequencing technologies for resolving highly complex microbiota composition using tandem variable 16S rRNA gene regions. Nucleic Acids Res 38: e200. 20880993 10.1093/nar/gkq873 PMC3001100

[52] Darfeuille-Michaud A, Boudeau J, Bulois P, Neut C, Glasser AL, et al (2004) High prevalence of adherent-invasive *Escherichia coli* associated with ileal mucosa in Crohn's disease. Gastroenterology 127: 412–421. 15300573 10.1053/j.gastro.2004.04.061

[53] Simpson KW, Dogan B, Rishniw M, Goldstein RE, Klaessig S, et al (2006) Adherent and invasive *Escherichia coli* is associated with granulomatous colitis in boxer dogs. Infect Immun 74: 4778–4792. 16861666 10.1128/IAI.00067-06 PMC1539603

[54] Abujamel T, Cadnum JL, Jury LA, Sunkesula VC, Kundrapu S, et al (2013) Defining the vulnerable period for re-establishment of *Clostridium difficile* colonization after treatment of *C. difficile* infection with oral vancomycin or metronidazole. PLOS ONE 8: e76269. 24098459 10.1371/journal.pone.0076269 PMC3788714

[55] Dion YM, Richards GK, Prentis JJ, Hinchey EJ (1980) The influence of oral versus parenteral preoperative metronidazole on sepsis following colon surgery. Ann Surg 192: 221–226. 6996626 10.1097/00000658-198008000-00016 PMC1344857

[56] Nitzan O, Elias M, Chazan B, Raz R, Saliba W (2013) *Clostridium difficile* and inflammatory bowel disease: role in pathogenesis and implications in treatment. World J Gastroenterol 19: 7577–7585. 24282348 10.3748/wjg.v19.i43.7577 PMC3837256

[57] Kanizaj TF, Kunac N (2014) Helicobacter pylori: Future perspectives in therapy reflecting three decades of experience. World J Gastroenterol 20: 699–705. 24574743 10.3748/wjg.v20.i3.699 PMC3921479

[58] Dethlefsen L, Huse S, Sogin ML, Relman DA (2008) The pervasive effects of an antibiotic on the human gut microbiota, as revealed by deep 16S rRNA sequencing. PLOS Biol 6: e280. 19018661 10.1371/journal.pbio.0060280 PMC2586385

[59] Dethlefsen L, Relman DA (2011) Incomplete recovery and individualized responses of the human distal gut microbiota to repeated antibiotic perturbation. Proc Natl Acad Sci USA 108: 4554–4561. 20847294 10.1073/pnas.1000087107 PMC3063582

[60] De La Cochetière MF, Durand T, Lepage P, Bourreille A, Galmiche JP, et al (2005) Resilience of the dominant human fecal microbiota upon short-course antibiotic challenge. J Clin Microbiol 43: 5588–5592. 16272491 10.1128/JCM.43.11.5588-5592.2005 PMC1287787

[61] Relman DA (2012) The human microbiome: ecosystem resilience and health. Nutr Rev 70: S2–S9. 22861804 10.1111/j.1753-4887.2012.00489.x PMC3422777

[62] McFarland LV (2006) Meta-analysis of probiotics for the prevention of antibiotic associated diarrhea and the treatment of *Clostridium difficile* disease. Am J Gastroenterol 101: 812–822. 16635227 10.1111/j.1572-0241.2006.00465.x

[63] Willard MD, Berridge B, Braniecki A, Bouley D (1998) Possible antibiotic-associated colitis in a dog. J Am Vet Med Assoc 213: 1775–1779. 9861973

[64] Ayyagari A, Agarwal J, Garg A (2003) Antibiotic associated diarrhoea: infectious causes. Indian J Med Microbiol 21: 6–11. 17642966

[65] Martin SJ, Yost RJ (2011) Infectious diseases in the critically ill patients. J Pharm Pract 24: 35–43. 21507873 10.1177/0897190010388906

[66] Sharma R, Sharma CL, Kapoor B (2005) Antibacterial resistance: current problems and possible solutions. Indian J Med Sci 59: 120–129. 15805685

[67] Malewska K, Rychlik A, Nieradka R, Kander M (2011) Treatment of inflammatory bowel disease (IBD) in dogs and cats. Pol J Vet Sci 14: 165–171. 21528730 10.2478/v10181-011-0026-7

[68] Cario E (2010) Toll-like receptors in inflammatory bowel diseases: a decade later. Inflamm Bowel Dis 16: 1583–1597. 20803699 10.1002/ibd.21282 PMC2958454

[69] Ivanov II, Atarashi K, Manel N, Brodie EL, Shima T, et al (2009) Induction of intestinal Th17 cells by segmented filamentous bacteria. Cell 139: 485–498. 19836068 10.1016/j.cell.2009.09.033 PMC2796826

[70] Kahn CE (2005) The Merck Veterinary Manual 9th ed. NJ: Merck & Co Whitehouse Station. 2128 p.

[71] Tsukamoto A, Ohno K, Maeda S, Nakashima K, Fukushima K, et al (2012) Effect of mosapride on prednisolone-induced gastric mucosal injury and gastric-emptying disorder in dog. J Vet Med Sci 74: 1103–1108. 22531101 10.1292/jvms.12-0066

[72] Garcia-Mazcorro JF, Dowd SE, Poulsen J, Steiner JM, Suchodolski JS (2012) Abundance and short-term temporal variability of fecal microbiota in healthy dogs. Microbiologyopen 1: 340–347. 23170232 10.1002/mbo3.36 PMC3496977

