# Supplementary figures and images for: Effect of Oral Administration of Metronidazole or Prednisolone on Fecal Microbiota in Dogs
Source: PLoS One. 2014 Sep 17;9(9):e107909. doi: 10.1371/journal.pone.0107909 (PMC4168260; doi:10.1371/journal.pone.0107909)

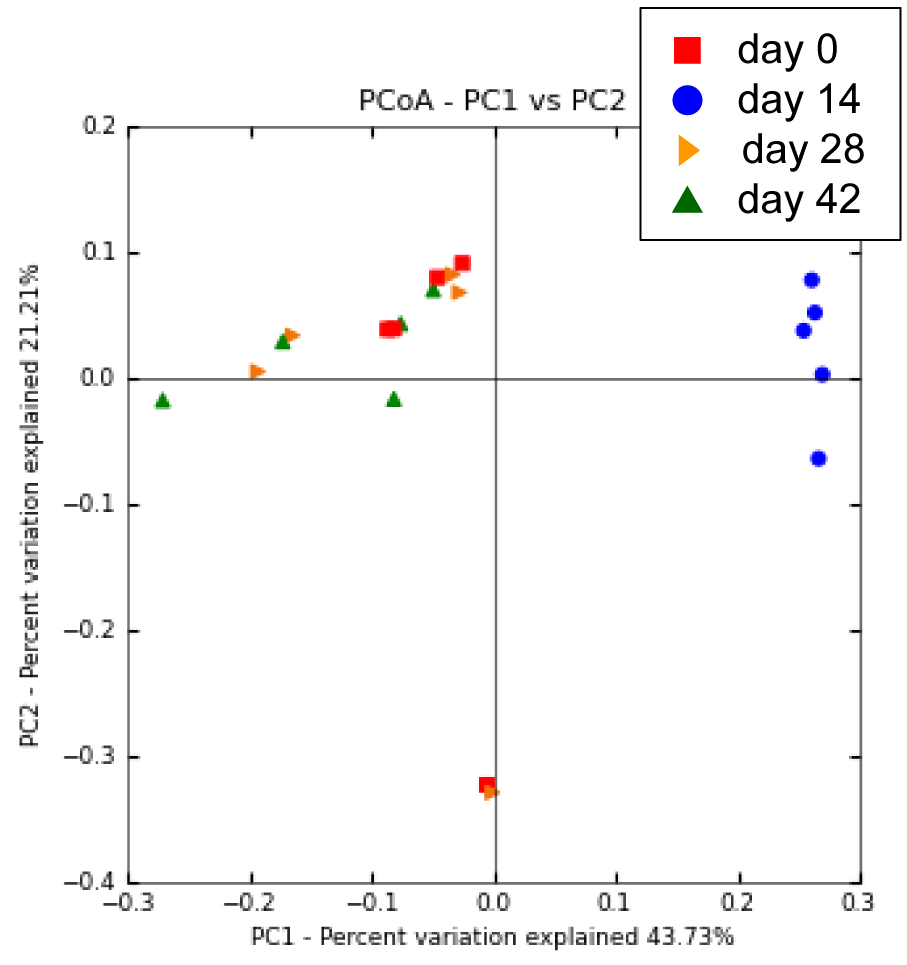

Supplement: Figure S1 — Principal coordinates analysis (PCoA) of weighted UniFrac distances of 16S rRNA genes in dogs administered metronidazole. Metronidazole-affected samples (blue, day 14) were separated from other samples, primarily along PCoA axis 1 (accounting for 43.73% of all variability among samples). (TIF) [file pone.0107909.s001.tif]

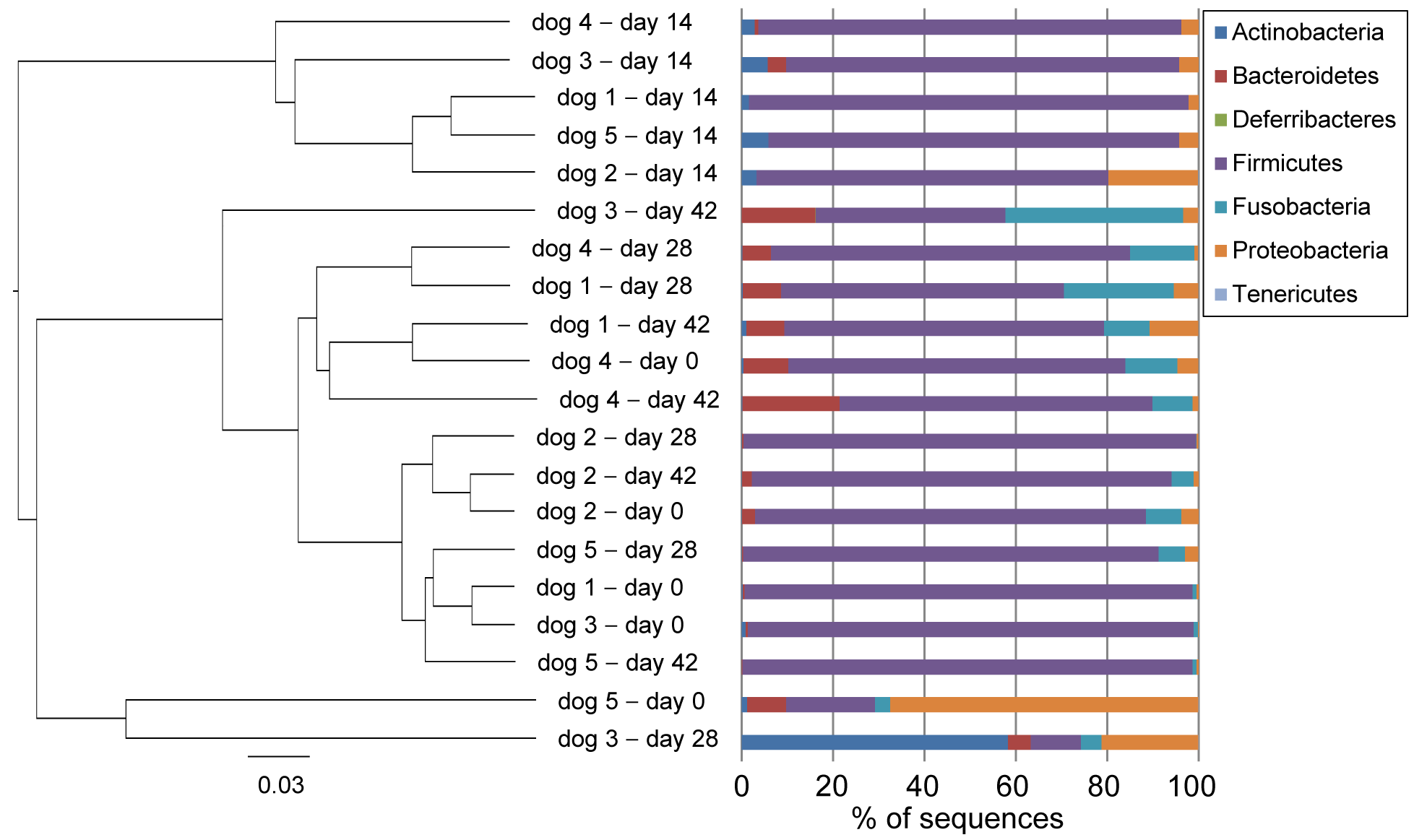

Supplement: Figure S2 — Hierarchical dendrogram based on weighted UniFrac distances of 16S rRNA genes and fecal microbial composition of each sample at phylum level in dogs administered metronidazole. This dendrogram showed that the samples obtained at day 14 were clustered. (TIF) [file pone.0107909.s002.tif]

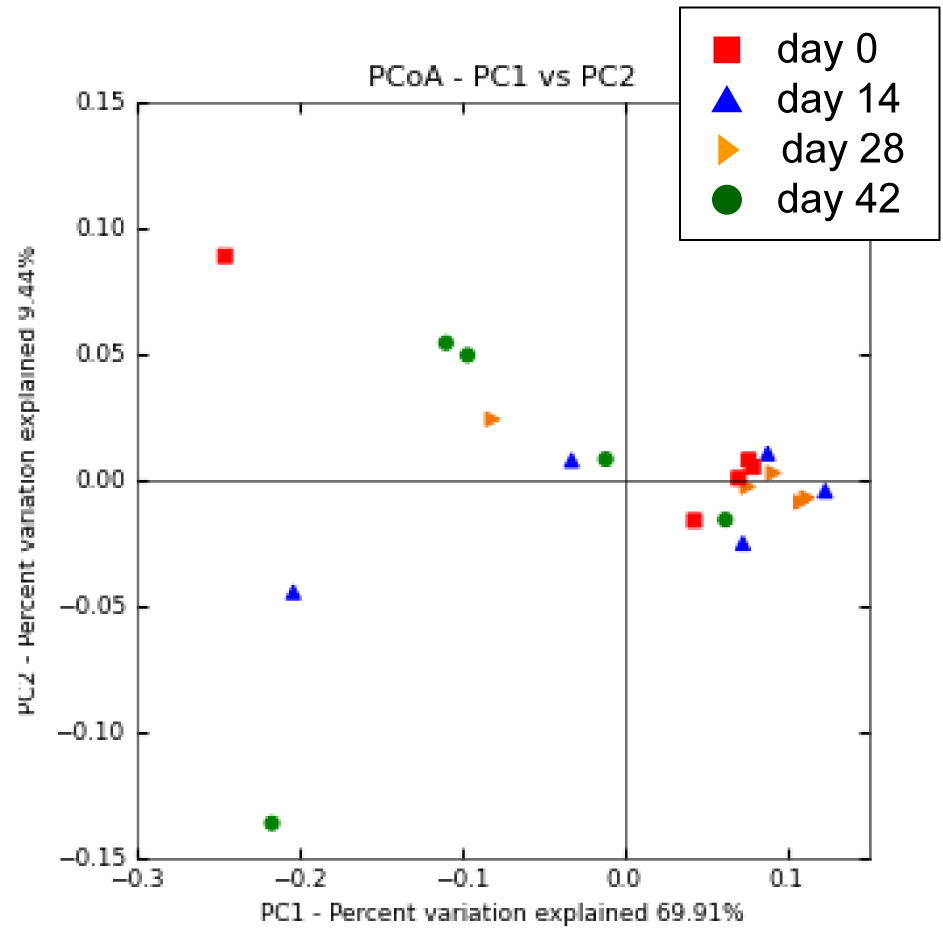

Supplement: Figure S3 — PCoA plots of weighted UniFrac distances of 16S rRNA genes in dogs administered prednisolone. No clustering was observed at any time points. (TIF) [file pone.0107909.s003.tif]

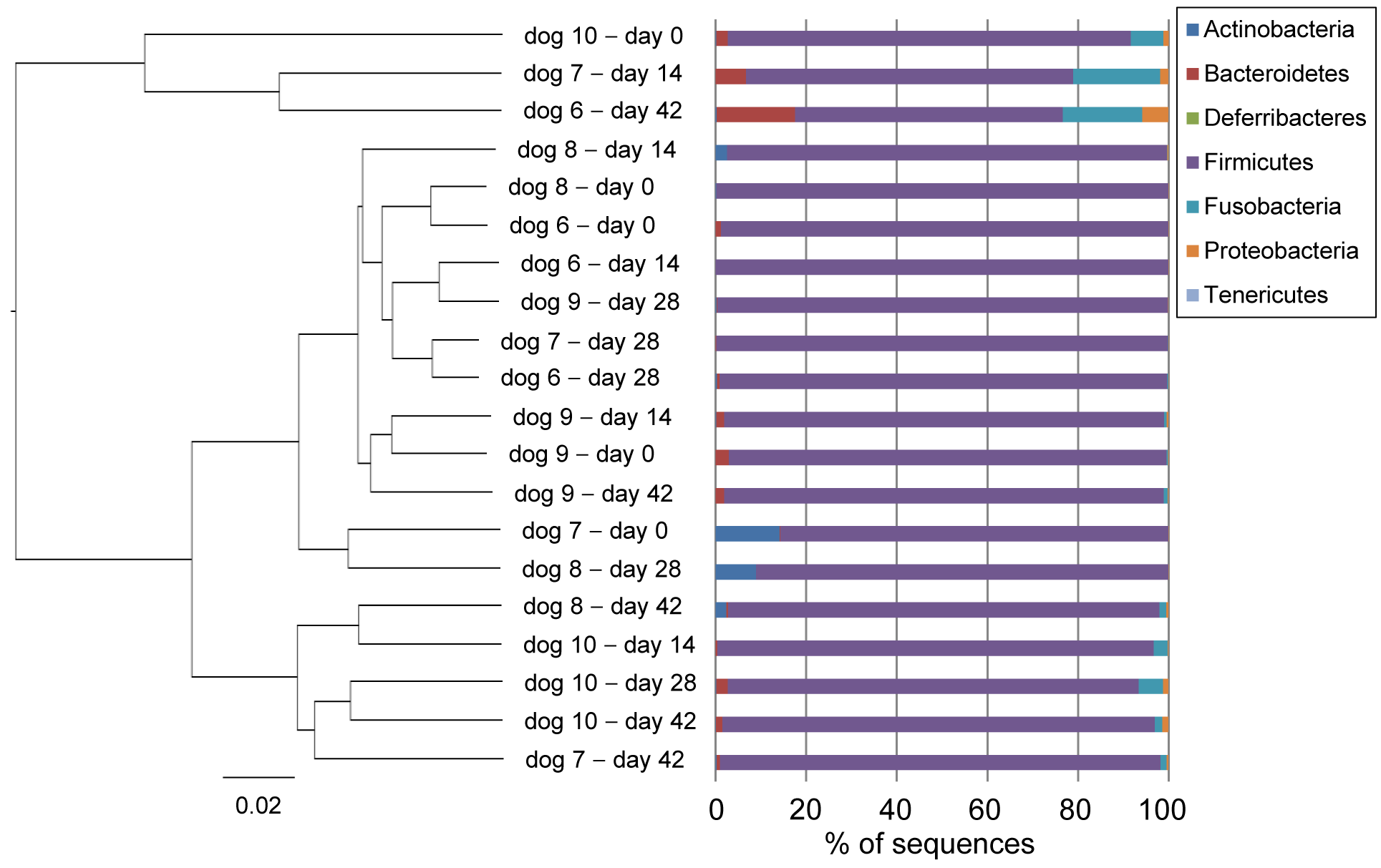

Supplement: Figure S4 — Hierarchical dendrogram based on weighted UniFrac distances of 16S rRNA genes and fecal microbial composition of each sample at phylum level in dogs administered prednisolone. This dendrogram showed that groups of samples at each time point were not clustered. (TIF) [file pone.0107909.s004.tif]
