# Supplementary material for: Effect of Oral Administration of Metronidazole or Prednisolone on Fecal Microbiota in Dogs
Source: PLoS One. 2014 Sep 17;9(9):e107909. doi: 10.1371/journal.pone.0107909 (PMC4168260; doi:10.1371/journal.pone.0107909)
Supplement: Table S1 — Relative proportions of bacterial taxa in dogs administered metronidazole. (PDF) [file pone.0107909.s005.pdf]

Table S1. Relative proportions of predominant bacterial taxa in the dogs with metronidazole administration

	Medians % (min.–max. %) of sequences			
	day 0	day 14	day 28	day 42
Actinobacteria (phylum)	0.38 (0.02–1.20)	3.19* (1.58–5.81)	0.17 (0.03–58.24)	0.03† (0.02–1.01)
Actinobacteria (class)	0.38 (0.02–1.20)	3.19* (1.58–5.81)	0.17 (0.03–58.24)	0.03† (0.02–1.01)
Actinomycetales	0.01 (0.00–0.45)	0.07 (0.02–0.10)	0.08 (0.03–58.21)	0.02 (0.00–0.17)
Actinomycetaceae	0.00 (0.00–0.02)	0.01 (0.00–0.02)	0.01 (0.00–0.03)	0.01 (0.00–0.08)
Actinomyces	0.00 (0.00–0.01)	0.01 (0.00–0.01)	0.01 (0.00–0.03)	0.01 (0.00–0.08)
Corynebacteriaceae	0.01 (0.00–0.39)	0.05 (0.02–0.08)	0.05 (0.01–58.07)	0.00 (0.00–0.07)
Corynebacterium	0.01 (0.00–0.39)	0.05 (0.02–0.08)	0.05 (0.01–58.07)	0.00 (0.00–0.07)
Microbacteriaceae	0.00 (0.00–0.01)	0.01 (0.00–0.03)	0.00 (0.00–0.01)	0.01 (0.00–0.01)
Leucobacter	0.00 (0.00–0.01)	0.01 (0.00–0.03)	0.00 (0.00–0.01)	0.01 (0.00–0.01)
Bifidobacteriales	0.38 (0.01–0.84)	3.18* (1.48–5.71)	0.03† (0.00–0.18)	0.02† (0.00–0.84)
Bifidobacteriaceae	0.37 (0.01–0.84)	3.18* (1.48–5.71)	0.03† (0.00–0.18)	0.02† (0.00–0.84)
Bifidobacterium	0.37 (0.01–0.84)	3.18* (1.48–5.71)	0.03† (0.00–0.18)	0.02† (0.00–0.84)
Bacteroidetes	2.84 (0.36–9.82)	0.02 (0.00–4.01)	4.95 (0.27–8.36)	8.27† (0.14–21.26)
Bacteroidia	2.84 (0.36–9.82)	0.02 (0.00–4.01)	4.95 (0.27–8.36)	8.27† (0.14–21.26)
Bacteroidales	2.84 (0.36–9.82)	0.02 (0.00–4.01)	4.95 (0.27–8.36)	8.27† (0.14–21.26)
Bacteroidaceae	1.93 (0.07–6.89)	0.01* (0.00–0.04)	1.39 (0.15–3.17)	5.01† (0.07–7.80)
Bacteroides	1.93 (0.07–6.89)	0.01* (0.00–0.04)	1.39 (0.15–3.17)	5.01† (0.07–7.80)
Porphyromonadaceae	0.01 (0.00–0.06)	0.00 (0.00–0.00)	0.00 (0.00–0.17)	0.02 (0.00–0.22)
Parabacteroides	0.01 (0.00–0.06)	0.00 (0.00–0.00)	0.00 (0.00–0.17)	0.02 (0.00–0.22)
Prevotellaceae	0.87 (0.26–6.91)	0.00 (0.00–4.00)	2.38 (0.04–4.98)	2.54 (0.07–15.80)
Prevotella	0.87 (0.26–6.91)	0.00 (0.00–4.00)	2.38 (0.04–4.98)	2.54 (0.07–15.80)
Paraprevotellaceae	0.04 (0.00–0.67)	0.00* (0.00–0.00)	0.05 (0.00–0.19)	0.12 (0.00–0.32)
S24-7	0.00 (0.00–0.01)	0.00 (0.00–0.01)	0.01 (0.00–0.02)	0.01 (0.00–0.09)
Firmicutes	85.61 (19.42–98.03)	89.92 (77.01–96.23)	78.59 (11.02–99.02)	69.97 (41.54–98.51)
Bacilli	4.66 (0.25–11.90)	68.75* (44.00–80.10)	1.36† (0.73–5.42)	3.66† (1.60–10.72)
Lactobacillales	0.29 (0.05–11.84)	68.74* (43.84–80.07)	0.07† (0.03–5.31)	0.12† (0.03–4.04)
Enterococcaceae	0.01 (0.00–10.59)	2.58 (1.07–8.20)	0.00 (0.00–4.70)	0.00 (0.00–0.01)
Enterococcus	0.00 (0.00–0.10)	0.11* (0.07–0.35)	0.00 (0.00–0.23)	0.00† (0.00–0.00)
Lactobacillaceae	0.28 (0.01–5.04)	10.13 (0.01–66.89)	0.04 (0.01–0.42)	0.05 (0.01–4.00)
Lactobacillus	0.13 (0.01–5.02)	6.31 (0.01–66.41)	0.01 (0.00–0.42)	0.02 (0.01–4.00)
Streptococcaceae	0.01 (0.00–0.17)	58.49* (0.69–71.24)	0.03† (0.01–0.13)	0.03† (0.02–0.06)
Lactococcus	0.00 (0.00–0.00)	0.01 (0.00–0.02)	0.00 (0.00–0.01)	0.00 (0.00–0.01)
Streptococcus	0.00 (0.00–0.13)	1.16* (0.05–1.61)	0.03 (0.00–0.12)	0.01† (0.01–0.03)
Turicibacterales	2.36 (0.04–5.76)	0.01* (0.00–1.17)	0.74 (0.12–1.73)	3.58† (1.45–6.66)
Turicibacteraceae	2.36 (0.04–5.76)	0.01* (0.00–1.17)	0.74 (0.12–1.73)	3.58† (1.45–6.66)
Turicibacter	2.36 (0.04–5.76)	0.01* (0.00–1.17)	0.74 (0.12–1.73)	3.58† (1.45–6.66)
Clostridia	74.50 (5.93–77.43)	3.45* (0.54–7.67)	65.34† (4.62–87.46)	48.75† (32.31–79.63)
Clostridiales	73.61 (5.90–76.87)	2.99* (0.46–7.59)	64.14† (4.59–87.09)	47.97† (32.09–78.95)
Clostridiaceae	40.44 (2.42–51.29)	1.25* (0.02–3.73)	35.99† (1.13–65.48)	20.86† (11.28–49.81)
Clostridium	38.73 (2.42–51.27)	0.13* (0.02–2.90)	35.90† (1.11–65.43)	20.52† (10.87–49.78)
Eubactericaea	2.30 (0.19–5.04)	1.28 (0.16–8.50)	0.83 (0.03–1.40)	1.07 (0.85–4.00)
Eubacterium	2.30 (0.19–5.04)	1.28 (0.16–8.50)	0.83 (0.03–1.40)	1.07 (0.85–4.00)
Lachnospiraceae	16.11 (1.63–18.98)	0.03* (0.01–0.05)	13.36 (1.37–20.28)	13.64† (9.01–19.60)
Blautia	11.01 (0.28–16.44)	0.02* (0.00–0.03)	5.08 (0.70–14.72)	9.21† (6.36–11.46)
Dorea	0.47 (0.10–2.57)	0.01* (0.00–0.01)	2.17 (0.02–11.93)	1.38† (0.68–5.11)
Roseburia	0.00 (0.00–0.09)	0.00 (0.00–0.00)	0.02 (0.00–0.26)	0.00 (0.00–0.01)
Peptococcaceae	2.44 (0.00–3.50)	0.00* (0.00–0.00)	0.12 (0.00–1.61)	0.52 (0.00–2.14)
Peptococcus	2.44 (0.00–3.50)	0.00* (0.00–0.00)	0.12 (0.00–1.61)	0.52 (0.00–2.14)
Peptostreptococcaceae	3.37 (0.21–5.85)	0.27 (0.10–4.27)	1.27 (0.32–4.40)	4.09 (1.59–6.61)
Peptostreptococcus	0.00 (0.00–0.13)	0.01 (0.00–0.02)	0.00 (0.00–0.82)	0.00 (0.00–0.01)

Table S1. Cont.

	Medians % (min.–max. %) of sequences			
	day 0	day 14	day 28	day 42
Ruminococcaceae	4.51 (0.81–10.16)	0.03* (0.00–0.32)	6.02 (0.97–8.05)	6.49† (5.13–20.97)
Faecalibacterium	0.19 (0.00–0.78)	0.00* (0.00–0.00)	0.17 (0.00–1.90)	0.76† (0.00–3.97)
Oscillospira	0.00 (0.00–0.09)	0.00 (0.00–0.00)	0.00 (0.00–0.01)	0.01 (0.00–0.05)
Ruminococcus	4.26 (0.60–9.97)	0.03* (0.00–0.32)	5.65 (0.76–6.24)	4.32† (2.68–20.86)
Veillonellaceae	0.12 (0.03–0.97)	0.00* (0.00–0.01)	0.40† (0.01–4.90)	1.45† (0.07–2.38)
Megamonas	0.04 (0.01–0.63)	0.00* (0.00–0.01)	0.38 (0.00–3.66)	0.88 (0.02–1.25)
Phascolarctobacterium	0.08 (0.02–0.60)	0.00* (0.00–0.00)	0.06 (0.01–1.18)	0.53† (0.03–0.70)
Coriobacteriales	0.29 (0.02–0.56)	0.18 (0.06–0.66)	0.48 (0.03–0.91)	0.41 (0.09–0.55)
Coriobacteriaceae	0.29 (0.02–0.56)	0.18 (0.06–0.66)	0.48 (0.03–0.91)	0.41 (0.09–0.55)
Adlercreutzia	0.01 (0.00–0.02)	0.00 (0.00–0.00)	0.00 (0.00–0.00)	0.01 (0.00–0.04)
Collinsella	0.26 (0.02–0.56)	0.18 (0.06–0.65)	0.44 (0.02–0.91)	0.38 (0.06–0.55)
Slackia	0.01 (0.00–0.03)	0.00 (0.00–0.02)	0.01 (0.00–0.05)	0.02 (0.00–0.03)
Erysipelotrichi	10.92 (0.67–20.97)	7.41 (2.93–40.69)	7.83 (0.96–22.78)	7.33 (4.70–26.00)
Erysipelotrichales	10.92 (0.67–20.97)	7.41 (2.93–40.69)	7.83 (0.96–22.78)	7.33 (4.70–26.00)
Erysipelotrichaceae	5.82 (0.46–20.80)	7.05 (2.93–39.39)	5.81 (0.95–17.69)	5.10 (2.98–25.47)
Allobaculum	5.74 (0.46–20.74)	6.75 (1.43–39.35)	5.61 (0.91–17.68)	4.64 (2.87–25.39)
Coprobacillaceae	2.65 (0.17–5.32)	0.35 (0.00–1.30)	2.02 (0.01–5.09)	1.71 (0.52–8.07)
Catenibacterium	2.37 (0.13–4.79)	0.33 (0.00–1.30)	1.68 (0.00–4.76)	1.64 (0.50–7.60)
Fusobacteria (phylum)	3.30 (0.88–11.40)	0.00* (0.00–0.01)	5.84† (0.13–23.98)	8.79† (0.87–38.85)
Fusobacteria (class)	3.30 (0.88–11.40)	0.00* (0.00–0.01)	5.84† (0.13–23.98)	8.79† (0.87–38.85)
Fusobacteriales	3.30 (0.88–11.40)	0.00* (0.00–0.01)	5.84† (0.13–23.98)	8.79† (0.87–38.85)
Fusobacteriaceae	3.30 (0.88–11.40)	0.00* (0.00–0.01)	5.84† (0.13–23.98)	8.79† (0.87–38.85)
Fusobacterium	0.00 (0.00–0.12)	0.00 (0.00–0.00)	0.00 (0.00–0.08)	0.01 (0.00–0.01)
J2-29	1.36 (0.01–1.79)	0.00* (0.00–0.00)	0.89 (0.01–2.40)	1.51† (0.03–13.05)
Proteobacteria	3.74 (0.11–67.45)	4.18 (2.15–19.64)	2.90 (0.41–21.19)	1.28 (0.41–10.72)
Betaproteobacteria	0.21 (0.09–3.24)	0.25 (0.06–1.16)	0.39 (0.00–1.62)	0.96 (0.03–1.95)
Burkholderiales	0.21 (0.09–3.24)	0.25 (0.06–1.16)	0.39 (0.00–1.62)	0.96 (0.03–1.95)
Alcaligenaceae	0.21 (0.09–3.24)	0.25 (0.06–1.16)	0.39 (0.00–1.62)	0.96 (0.03–1.95)
Sutterella	0.21 (0.09–3.24)	0.25 (0.06–1.16)	0.39 (0.00–1.62)	0.96 (0.03–1.95)
Epsilonproteobacteria	0.00 (0.00–21.30)	0.03 (0.00–0.22)	0.09 (0.00–19.29)	0.02 (0.00–1.84)
Campylobacterales	0.00 (0.00–21.30)	0.03 (0.00–0.22)	0.09 (0.00–19.29)	0.02 (0.00–1.84)
Helicobacteraceae	0.00 (0.00–21.30)	0.03 (0.00–0.22)	0.09 (0.00–19.29)	0.02 (0.00–1.84)
Helicobacter	0.00 (0.00–21.27)	0.03 (0.00–0.22)	0.09 (0.00–19.26)	0.02 (0.00–1.83)
Gammaproteobacteria	1.38 (0.02–45.79)	3.93 (1.96–18.26)	1.20 (0.18–3.71)	0.70 (0.30–6.94)
Aeromonadales	1.38 (0.00–40.83)	0.35 (0.02–14.51)	0.43 (0.17–3.54)	0.38 (0.07–6.84)
Succinivibrionaceae	1.38 (0.00–40.83)	0.35 (0.02–14.51)	0.43 (0.17–3.54)	0.38 (0.07–6.84)
Anaerobiospirillum	1.21 (0.00–40.39)	0.33 (0.02–14.51)	0.22 (0.00–3.52)	0.38 (0.00–6.80)
Enterobacteriales	0.00 (0.00–3.42)	3.58* (1.16–4.10)	0.02 (0.00–0.17)	0.00 (0.00–0.59)
Enterobacteriaceae	0.00 (0.00–3.42)	3.58* (1.16–4.10)	0.02 (0.00–0.17)	0.00 (0.00–0.59)
Escherichia	0.00 (0.00–0.47)	2.78* (0.97–3.54)	0.01 (0.00–0.17)	0.00† (0.00–0.53)
Morganella	0.00 (0.00–0.00)	0.14* (0.04–0.94)	0.00† (0.00–0.00)	0.00† (0.00–0.00)
Proteus	0.00 (0.00–2.93)	0.24 (0.09–0.52)	0.00† (0.00–0.00)	0.00† (0.00–0.02)
Pseudomonadales	0.00 (0.00–1.09)	0.00 (0.00–0.01)	0.00 (0.00–0.75)	0.03 (0.00–0.07)
Moraxellaceae	0.00 (0.00–0.12)	0.00 (0.00–0.01)	0.00 (0.00–0.05)	0.01 (0.00–0.07)
Psychrobacter	0.00 (0.00–0.03)	0.00 (0.00–0.00)	0.00 (0.00–0.04)	0.01 (0.00–0.07)

Taxa present in at least 3 of 5 dogs (either day 0, 14, 28, or 42) were included in this analysis.

*Significantly different from day 0 ($P < 0.05$).

†Significantly different from day 14 ($P < 0.05$).
