# Supplementary material for: Effect of Oral Administration of Metronidazole or Prednisolone on Fecal Microbiota in Dogs
Source: PLoS One. 2014 Sep 17;9(9):e107909. doi: 10.1371/journal.pone.0107909 (PMC4168260; doi:10.1371/journal.pone.0107909)
Supplement: Table S2 — Relative proportions of bacterial taxa in dogs administered prednisolone. (PDF) [file pone.0107909.s006.pdf]

Table S2. Relative proportions of bacterial taxa in dogs administered prednisolone

	Medians % (min.–max. %) of sequences			
	day 0	day 14	day 28	day 42
Actinobacteria (phylum)	0.16 (0.00–14.18)	0.02 (0.00–2.53)	0.24 (0.04–8.93)	0.23 (0.03–2.35)
Actinobacteria (class)	0.16 (0.00–14.18)	0.02 (0.00–2.53)	0.24 (0.04–8.93)	0.23 (0.03–2.35)
Actinomycetales	0.00 (0.00–0.14)	0.00 (0.00–0.01)	0.00 (0.00–0.29)	0.01 (0.00–0.30)
Actinomycetaceae	0.00 (0.00–0.11)	0.00 (0.00–0.01)	0.00 (0.00–0.01)	0.01 (0.00–0.03)
Bifidobacteriales	0.03 (0.00–14.17)	0.01 (0.00–2.53)	0.17 (0.04–8.93)	0.08 (0.00–2.35)
Bifidobacteriaceae	0.03 (0.00–14.11)	0.01 (0.00–2.50)	0.17 (0.04–8.90)	0.08 (0.00–2.35)
Bifidobacterium	0.03 (0.00–14.11)	0.01 (0.00–2.50)	0.17 (0.04–8.90)	0.08 (0.00–2.35)
Bacteroidetes	1.21 (0.04–2.95)	0.39 (0.01–6.69)	0.15 (0.05–2.51)	1.44 (0.39–17.22)
Bacteroidia	1.21 (0.04–2.95)	0.39 (0.01–6.69)	0.15 (0.05–2.51)	1.44 (0.39–17.22)
Bacteroidales	1.21 (0.04–2.95)	0.39 (0.01–6.69)	0.15 (0.05–2.51)	1.44 (0.39–17.22)
Bacteroidaceae	0.41 (0.02–2.30)	0.09 (0.00–3.12)	0.05 (0.01–2.09)	0.49 (0.12–7.29)
Bacteroides	0.41 (0.02–2.30)	0.09 (0.00–3.12)	0.05 (0.01–2.09)	0.49 (0.12–7.29)
Porphyromonadaceae	0.01 (0.00–0.11)	0.01 (0.00–0.05)	0.00 (0.00–0.02)	0.00 (0.00–0.04)
Parabacteroides	0.01 (0.00–0.09)	0.01 (0.00–0.04)	0.00 (0.00–0.01)	0.00 (0.00–0.04)
Prevotellaceae	0.26 (0.01–0.80)	0.23 (0.01–3.33)	0.12 (0.03–0.32)	0.57 (0.11–2.89)
Prevotella	0.26 (0.01–0.80)	0.23 (0.01–3.33)	0.12 (0.03–0.32)	0.57 (0.11–2.89)
Paraprevotellaceae	0.01 (0.00–0.09)	0.01 (0.00–0.13)	0.01 (0.00–0.18)	0.02 (0.00–7.03)
S24-7	0.00 (0.00–0.01)	0.00 (0.00–0.05)	0.00 (0.00–0.01)	0.01 (0.00–0.04)
Firmicutes	96.55 (85.58–99.66)	97.06 (72.22–99.93)	98.72 (90.65–99.70)	95.35 (59.13–97.19)
Bacilli	80.42 (17.00–91.78)	83.22 (25.60–97.36)	86.38 (56.37–92.54)	52.32 (19.21–85.36)
Bacillales	0.01 (0.00–0.04)	0.00 (0.00–0.00)	0.01 (0.00–0.01)	0.01 (0.00–0.20)
Staphylococcaceae	0.01 (0.00–0.04)	0.00 (0.00–0.00)	0.01 (0.00–0.01)	0.01 (0.00–0.20)
Gemellales	0.00 (0.00–0.04)	0.00 (0.00–0.00)	0.00 (0.00–0.06)	0.01 (0.00–0.01)
Gemellaceae	0.00 (0.00–0.04)	0.00 (0.00–0.00)	0.00 (0.00–0.06)	0.01 (0.00–0.01)
Gemella	0.00 (0.00–0.04)	0.00 (0.00–0.00)	0.00 (0.00–0.06)	0.01 (0.00–0.01)
Lactobacillales	80.38 (2.97–91.71)	83.07 (24.58–96.98)	86.35 (54.93–92.52)	48.52 (18.35–85.23)
Enterococcaceae	0.01 (0.00–0.34)	0.01 (0.00–0.02)	0.02 (0.02–0.07)	0.03 (0.00–0.34)
Lactobacillaceae	80.34 (0.87–91.61)	82.94 (24.56–96.62)	86.21 (54.08–92.31)	39.02 (17.86–85.05)
Lactobacillus	31.07 (0.14–61.25)	45.04 (2.69–76.63)	52.19 (1.84–78.67)	16.13 (0.69–44.68)
Streptococcaceae	0.08 (0.01–1.75)	0.06 (0.01–4.84)	0.17 (0.02–0.80)	0.11 (0.06–10.13)
Lactococcus	0.01 (0.00–0.11)	0.01 (0.00–0.02)	0.01 (0.00–0.01)	0.00 (0.00–0.02)
Streptococcus	0.04 (0.01–1.10)	0.01 (0.00–0.19)	0.03 (0.00–0.15)	0.06 (0.02–0.69)
Turicibacterales	0.19 (0.03–13.92)	0.16 (0.01–1.00)	0.04 (0.01–1.37)	0.62 (0.08–3.76)
Turicibacteraceae	0.19 (0.03–13.92)	0.16 (0.01–1.00)	0.04 (0.01–1.37)	0.62 (0.08–3.76)
Turicibacter	0.19 (0.03–13.92)	0.16 (0.01–1.00)	0.04 (0.01–1.37)	0.62 (0.08–3.76)
Clostridia	13.16 (3.40–66.03)	11.57 (1.50–43.22)	9.34 (5.25–30.95)	36.93 (10.13–48.75)
Clostridiales	12.86 (3.26–64.48)	11.20 (1.44–30.65)	9.16 (5.05–30.31)	36.65 (10.03–48.10)
Clostridiaceae	6.48 (0.80–27.10)	6.35 (0.66–20.61)	3.30 (2.90–14.02)	20.53 (4.88–21.49)
Clostridium	6.45 (0.79–25.82)	6.34 (0.65–20.47)	3.29 (2.86–13.78)	20.27 (4.88–20.97)
Eubacteriaceae	0.24 (0.10–0.97)	0.16 (0.09–0.66)	0.36 (0.04–0.53)	0.23 (0.10–0.69)
Eubacterium	0.24 (0.10–0.95)	0.16 (0.09–0.66)	0.36 (0.04–0.53)	0.23 (0.10–0.68)
Lachnospiraceae	2.77 (0.82–14.75)	2.23 (0.57–13.42)	1.83 (0.96–9.09)	5.38 (1.70–13.00)
Blautia	1.86 (0.59–5.67)	1.21 (0.49–10.42)	1.25 (0.61–6.13)	2.39 (1.06–9.39)
Dorea	0.33 (0.05–5.26)	0.44 (0.04–1.01)	0.14 (0.07–1.25)	1.07 (0.21–2.19)
Peptococcaceae	0.67 (0.00–2.95)	0.45 (0.03–3.35)	0.25 (0.10–1.91)	0.47 (0.02–2.29)
Peptococcus	0.67 (0.00–2.95)	0.45 (0.03–3.35)	0.25 (0.10–1.91)	0.47 (0.02–2.29)
Peptostreptococcaceae	0.08 (0.00–1.54)	0.02 (0.00–2.29)	0.01 (0.00–0.57)	0.29 (0.05–7.70)
Ruminococcaceae	1.39 (0.41–13.66)	1.83 (0.13–4.59)	0.98 (0.49–3.26)	3.26 (1.30–4.34)
Faecalibacterium	0.04 (0.00–0.11)	0.04 (0.00–0.64)	0.08 (0.00–0.13)	0.01 (0.00–0.34)
Ruminococcus	1.31 (0.41–13.26)	1.71 (0.13–3.84)	0.91 (0.48–3.10)	3.23 (1.29–3.97)
Veillonellaceae	0.74 (0.01–4.41)	0.46 (0.03–1.99)	1.44 (0.01–4.27)	2.74 (0.29–6.36)
Megamonas	0.49 (0.00–3.65)	0.04 (0.01–1.76)	0.14 (0.01–1.06)	0.95 (0.19–5.67)
Megasphaera	0.00 (0.00–0.17)	0.02 (0.00–0.77)	0.00 (0.00–4.09)	0.00 (0.00–2.74)
Phascolarctobacterium	0.08 (0.00–0.48)	0.01 (0.00–0.25)	0.01 (0.00–0.24)	0.05 (0.02–0.59)
Coriobacteriales	0.19 (0.14–1.41)	0.17 (0.03–0.29)	0.16 (0.12–0.58)	0.24 (0.06–0.95)
Coriobacteriaceae	0.19 (0.14–1.41)	0.17 (0.03–0.29)	0.16 (0.12–0.58)	0.24 (0.06–0.95)
Collinsella	0.19 (0.14–1.35)	0.16 (0.03–0.22)	0.16 (0.12–0.57)	0.22 (0.06–0.95)
Slackia	0.00 (0.00–0.06)	0.01 (0.00–0.04)	0.00 (0.00–0.01)	0.02 (0.00–0.03)

Table S2. Cont.

	Medians % (min.–max. %) of sequences			
	day 0	day 14	day 28	day 42
Erysipelotrichi	2.55 (1.77–4.90)	1.48 (0.88–6.99)	1.57 (0.95–2.74)	2.77 (1.33–7.54)
Erysipelotrichales	2.55 (1.77–4.90)	1.48 (0.88–6.99)	1.57 (0.95–2.74)	2.77 (1.33–7.54)
Erysipelotrichaceae	1.70 (0.38–3.54)	1.37 (0.23–6.68)	0.79 (0.43–2.21)	2.51 (0.98–4.00)
Allobaculum	1.27 (0.37–3.37)	1.33 (0.23–6.59)	0.76 (0.42–2.14)	2.50 (0.95–3.83)
Coprobacillaceae	0.94 (0.05–2.17)	0.11 (0.02–0.65)	0.66 (0.36–1.04)	0.46 (0.26–4.58)
Catenibacterium	0.94 (0.04–2.17)	0.11 0.01–0.64)	0.65 (0.36–1.03)	0.45 (0.26–4.58)
Fusobacteria (phylum)	0.01 (0.01–7.19)	0.51 (0.00–19.21)	0.06 (0.01–5.38)	1.53 (0.83–17.48)
Fusobacteria (class)	0.01 (0.01–7.19)	0.51 (0.00–19.21)	0.06 (0.01–5.38)	1.53 (0.83–17.48)
Fusobacteriales	0.01 (0.01–7.19)	0.51 (0.00–19.21)	0.06 (0.01–5.38)	1.53 (0.83–17.48)
Fusobacteriaceae	0.01 (0.01–7.19)	0.51 (0.00–19.21)	0.06 (0.01–5.38)	1.53 (0.83–17.48)
Fusobacterium	0.01 (0.00–0.04)	0.01 (0.00–0.02)	0.00 (0.00–0.04)	0.00 (0.00–0.01)
J2-29	0.00 (0.00–0.77)	0.03 (0.00–2.92)	0.01 (0.00–1.88)	0.15 (0.07–4.02)
Proteobacteria	0.08 (0.04–1.18)	0.29 (0.03–1.77)	0.04 (0.02–1.23)	0.50 (0.20–5.82)
Betaproteobacteria	0.01 (0.01–0.34)	0.06 (0.00–0.58)	0.01 (0.00–0.71)	0.18 (0.01–0.32)
Burkholderiales	0.01 (0.01–0.34)	0.06 (0.00–0.58)	0.01 (0.00–0.71)	0.18 (0.01–0.32)
Alcaligenaceae	0.01 (0.01–0.34)	0.06 (0.00–0.57)	0.01 (0.00–0.71)	0.18 (0.01–0.32)
Sutterella	0.01 (0.01–0.34)	0.06 (0.00–0.57)	0.01 (0.00–0.71)	0.18 (0.01–0.32)
Gammaproteobacteria	0.08 (0.03–0.64)	0.16 (0.03–1.17)	0.02 (0.02–0.49)	0.22 (0.17–5.73)
Aeromonadales	0.03 (0.00–0.27)	0.15 (0.00–1.08)	0.01 (0.00–0.38)	0.13 (0.07–0.95)
Succinivibrionaceae	0.03 (0.00–0.27)	0.15 (0.00–1.08)	0.01 (0.00–0.38)	0.13 (0.07–0.95)
Anaerobiospirillum	0.00 (0.00–0.27)	0.15 (0.00–1.08)	0.01 (0.00–0.38)	0.06 (0.00–0.95)
Enterobacteriales	0.04 (0.00–0.08)	0.07 (0.01–0.10)	0.02 (0.00–0.05)	0.11 (0.06–5.56)
Enterobacteriaceae	0.04 (0.00–0.08)	0.07 (0.01–0.10)	0.02 (0.00–0.05)	0.11 (0.06–5.56)
Escherichia	0.04 (0.00–0.08)	0.06 (0.01–0.08)	0.01 (0.00–0.05)	0.11 (0.05–0.15)

Taxa present in at least three of five dogs (day 0, 14, 28, or 42) were included in this analysis.
